# How Molecular Competition Influences Fluxes in Gene Expression Networks

**DOI:** 10.1371/journal.pone.0028494

**Published:** 2011-12-05

**Authors:** Dirk De Vos, Frank J. Bruggeman, Hans V. Westerhoff, Barbara M. Bakker

**Affiliations:** 1 Molecular Cell Physiology, Netherlands Institute for Systems Biology, Department of Molecular Cell Biology, Vrije Universiteit Amsterdam, Amsterdam, The Netherlands; 2 Kluyver Centre for Genomics of Industrial Fermentation, Delft, The Netherlands; 3 Regulatory Networks Group, Netherlands Institute for Systems Biology, Life Sciences, Centre for Mathematics and Computer Science, Amsterdam, The Netherlands; 4 Manchester Centre for Integrative Systems Biology, Manchester Interdisciplinary Biocentre, the University of Manchester, Manchester, United Kingdom; 5 Department of Pediatrics, Center for Liver, Digestive and Metabolic Diseases, University Medical Center Groningen, University of Groningen, Groningen, The Netherlands; Semmelweis University, Hungary

## Abstract

Often, in living cells different molecular species compete for binding to the same molecular target. Typical examples are the competition of genes for the transcription machinery or the competition of mRNAs for the translation machinery. Here we show that such systems have specific regulatory features and how they can be analysed. We derive a theory for molecular competition in parallel reaction networks. Analytical expressions for the response of network fluxes to changes in the total competitor and common target pools indicate the precise conditions for ultrasensitivity and intuitive rules for competitor strength. The calculations are based on measurable concentrations of the competitor-target complexes. We show that kinetic parameters, which are usually tedious to determine, are not required in the calculations. Given their simplicity, the obtained equations are easily applied to networks of any dimension. The new theory is illustrated for competing sigma factors in bacterial transcription and for a genome-wide network of yeast mRNAs competing for ribosomes. We conclude that molecular competition can drastically influence the network fluxes and lead to negative response coefficients and ultrasensitivity. Competitors that bind a large fraction of the target, like bacterial σ^70^, tend to influence competing pathways strongly. The less a competitor is saturated by the target, the more sensitive it is to changes in the concentration of the target, as well as to other competitors. As a consequence, most of the mRNAs in yeast turn out to respond ultrasensitively to changes in ribosome concentration. Finally, applying the theory to a genome-wide dataset we observe that high and low response mRNAs exhibit distinct Gene Ontology profiles.

## Introduction

One of the main challenges of current systems biology is to understand the properties of large and complex molecular networks [1], [2]. With increasingly more genome-scale datasets becoming available, the need to interpret them from a mechanistic perspective rather than in a purely descriptive manner is growing. We have developed a theoretical framework that addresses this need, for a universal type of network structure found in transcription and translation as well as in other molecular processes.

It is expected that natural selection minimizes the cost of producing abundant catalytic machinery, for example the large ribosomal complexes involved in translation or RNA polymerase in transcription [3]–[5]. Reduction in these pools leads to enhanced competition between distinct binding partners. Competition between mRNA species for ribosomes is therefore important to take into consideration. Computational modelling of large-scale translation networks has demonstrated that system-wide competition for ribosomes results in nonlinear effects that can have significant impact on the interpretation of the relationship between mRNA levels and protein expression [6].

**Figure 1:**
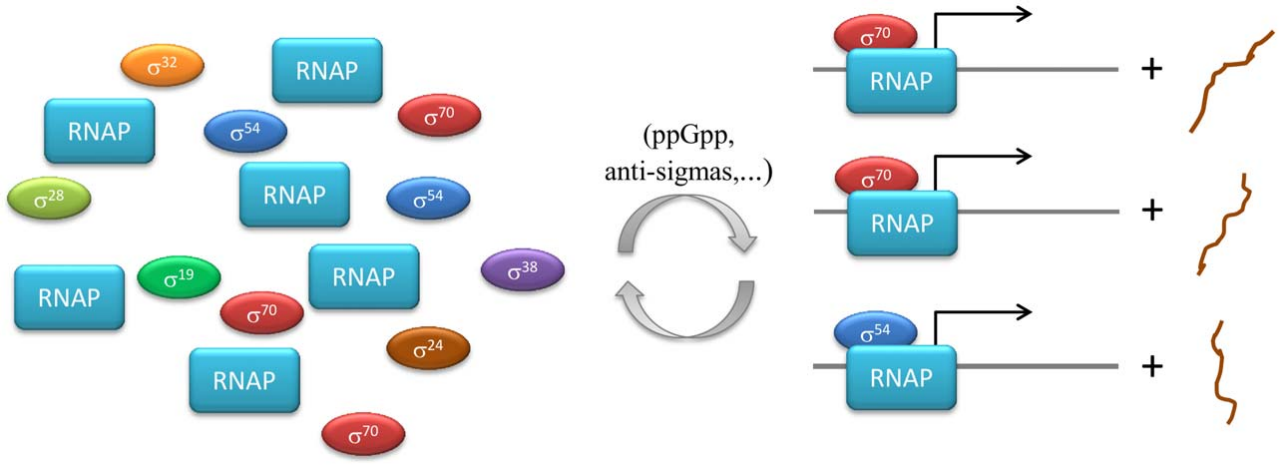
Sigma cycle in *E. Coli*. The sigma cycle in bacterial transcription refers to gene regulation by competitive association of promoter-specific transcription factors -sigma factors- with RNA polymerase (RNAP; [7]–[9]). The sigma factors compete for binding to RNAP after each round of RNA synthesis, and determine the promoter-specificity of transcription initiation (see for instance [7] for more mechanistic detail). In *E. coli*, seven different types of sigma factors exist, each directing transcription of a specific set of genes [10]. Most of the growth-related and housekeeping genes expressed in the exponential phase of the growth of a cell population are transcribed by the RNAP-holoenzyme containing σ^70^ (σ^D^). σ^54^ (σ^N^) confers specificity to RNAP for transcribing genes regulated by the availability of nitrogen and some stress response genes [11], [12]. σ^28^ (σ^F^) is needed for the expression of the flagellum and chemotaxis genes [13]. σ^38^ (σ^S^) accumulates during the stationary phase and directs expression of genes related to stress management and maintenance [8]. The other three sigma factors, σ^32^ (σ^H^), σ^24^ (σ^E^) and σ^19^ (σ^FecI^), act in heat shock response, extra- cytoplasmic stress and iron-transport respectively [14]. Although other regulatory factors play a role (the alarmone ppGpp, anti-sigma factors, *etc.*; *cf.* [9]), the global pattern of gene transcription is believed to be determined largely through sigma factor competition [10].

Another example of molecular competition is the so-called sigma cycle in bacterial transcription (Figure 1). This type of gene regulation occurs by the competitive association of promoter-specific transcription factors -sigma factors- with RNA polymerase (RNAP; [7]–[9]). The sigma factors compete for binding to RNAP after each round of RNA synthesis, and determine the promoter-specificity of transcription initiation. In *E. coli*, seven different types of sigma factors exist, each directing transcription of a specific set of genes [10]. Most of the growth-related and housekeeping genes expressed in the exponential phase of the growth of a cell population are transcribed by the RNAP-holoenzyme containing σ^70^. σ^54^ (also called σ^N^) confers specificity to RNAP for transcribing genes regulated by the availability of nitrogen and some stress response genes [11], [12]. σ^28^ (or σ^F^) is needed for the expression of the flagellum and chemotaxis genes [13]. σ^38^ (σ^S^) accumulates during the stationary phase and directs expression of genes related to stress management and maintenance [8]. The other three sigma factors, σ^H^, σ^E^ and σ^FecI^, act in heat shock response, extra- cytoplasmic stress and iron-transport respectively [14]. Although other regulatory factors play a role (*e.g.* [9]), the global pattern of gene transcription is believed to be determined largely through sigma factor competition [10]. Therefore, the competition between the various sigma factors for RNAP is essential to our understanding of bacterial adaptation in different conditions.

To understand how strongly regulation in these and analogous networks is affected by molecular competition, we here develop a general theoretical framework. Based on the theory of Metabolic Control Analysis [15]–[17], we derive formulas to calculate so-called response (or sensitivity) coefficients, which express the fractional change in some variable (*e.g.* flux) towards a small fractional change in an external parameter (in this case the total concentration of the target or a specific competitor). For instance, if a specific translation flux depends proportionally on the specific mRNA concentration, then the corresponding response coefficient 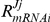 equals 1. If that translation flux is negatively affected by increasing a competing mRNA concentration, this leads to a negative response coefficient. In the special case of an ultrasensitive (sigmoidal) dependence of the flux towards an mRNA concentration, the corresponding response coefficient exceeds 1 over a range of mRNA concentrations. The maximum response coefficient attained then equals the so-called Hill-coefficient, another measure of cooperativity or ultrasensitivity [18].

The response coefficient corresponds to the infinitesimal form of the classical ‘amplification factor’ used by Goldbeter and Koshland to describe an input-output (stimulus-response) relation in any biochemical system [18]:



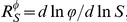
with *ϕ* the response and *S* the stimulus.

The finite version ([18]: equation (2) within),



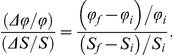
(with *i* and *f* indicating initial and final values, respectively) is less suitable than the continuous version to derive generic formula, given the nonlinear dependence on the size of the variation in stimulus.

**Figure 2:**
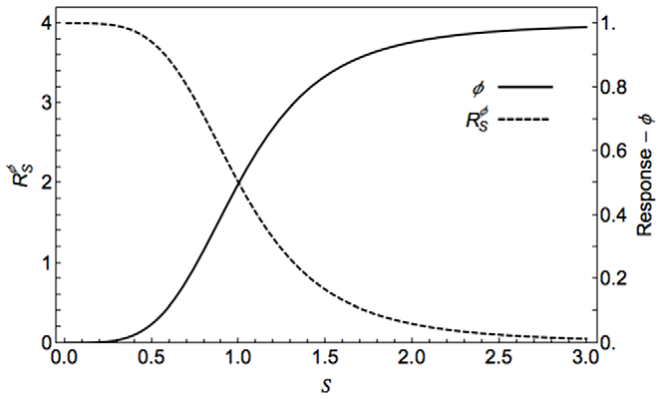
Relation between a sigmoidal (Hill type) input-output relation and the corresponding response coefficient. The reaction rate normalized to 1 (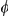) and the response coefficient towards a change in substrate concentration S (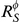) are plotted as a function of the substrate concentration (assuming excess substrate over enzyme).

Figure 2 illustrates the relation between a sigmoidal (Hill type) input-output relation and the corresponding response coefficient.

In the following we will specifically focus on the sensitivity of the steady state fluxes of the competing pathways towards changes in the levels of the competitors, as well as towards changes in the levels of the target for which they compete. We will first derive the general theory that allows for quantification of response coefficients based on *measurable* concentrations of the target-competitor complexes. This will be much more convenient than a classical, direct measurement of response coefficients, which requires a tedious titration of specific macromolecules, such as ribosomes, mRNAs or sigma factors. The resulting equations are analyzed and applied to both a small-scale and large-scale network: the sigma cycle and translation, respectively. This yields new insights in the regulation of these networks.

## Results

### Analytical theory: molecular competition for binding to a common target

**Figure 3:**
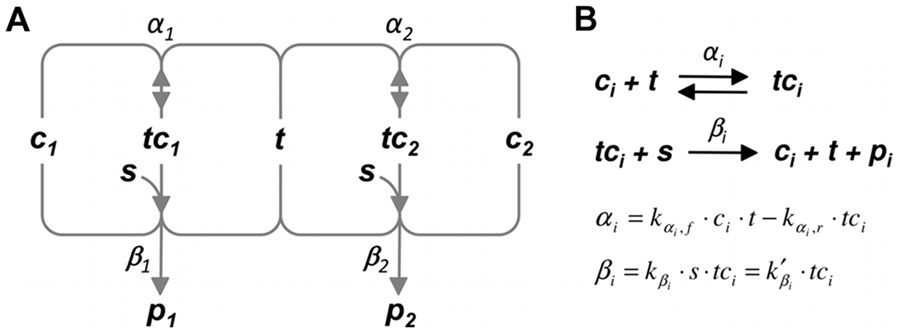
Basic network diagram of two molecules competing for a common target molecule. (A). The reaction scheme. In the reversible binding steps (with rates *α_1_* and *α_2_*) the free competitors *c_1_* and *c_2_* form a complex with the target *t* (to produce complexes *tc_1_* and *tc_2_*, respectively). In the irreversible production steps (with rates *β_1_* and *β_2_*) the products (*p_1_* and *p_2_*) are generated and the free competitor and the target are recycled. In the analogy to the bacterial transcription network the competitors are the sigma factors and the target is RNA polymerase. In the analogy to the translation network the competitors are the mRNAs and the target is the ribosome.(B). The reactions and rate equations for competitor *i*. The assumptions underlying these equations are discussed in the Introduction.

To explain the basic structure of the type of networks considered in our analysis we first turn to the most simple reaction scheme with two competitors *c_1_* and *c_2_*, and their common target *t* (Figure 3A). In the case of the sigma cycle in bacterial transcription the competitors are the sigma factors. The target then represents the pool of RNA polymerase for which they compete. Through reversible binding reactions the competitors form complexes with their target at rates *α_1_* and *α_2_*, respectively. In a second lumped reaction, these complexes convert the substrate *s* (*e.g.* nucleotides) at rates *β_1_* and *β_2_* into the products (*p_1_* and *p_2_*, respectively, *e.g.* for different mRNAs) and the products dissociate from the complex. Simultaneously the competitors are released from the target. The case of two competitors is generalized below to any number of competitors ‘*n*’, and to simultaneous binding of multiple identical target molecules to one competitor. The latter applies to translation, when a single mRNA (competitor) binds multiple ribosomes (the targets). In Figure 3B, the rate equations of the model with *n* competitors for binding to a single pool of target molecule are listed. The following assumptions are made:

1. The binding of the competitor to the target is described by reversible mass-action kinetics,
2. The subsequent production step is described by irreversible mass-action kinetics,
3. We assume the substrate concentration *s* to be constant and subsume it into the rate constant of the production reaction to yield an apparent rate constant 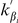.

The irreversibility of the production step (assumption 2) is warranted by the free-energy requirement of the elongation steps in transcription and translation, for which we have tailored the model. If we would explicitly consider reversible binding of the substrate (*e.g.* nucleotides) prior to its conversion to product (*e.g.* mRNA), the rates *β* would be better described by Michaelis-Menten equations, with *tc_1_* and *tc_2_* as the enzyme concentrations. Also then, the rates *β* remain proportional to *tc_1_* and *tc_2_*. We should be aware that a hyperbolic substrate function is then hidden in the apparent reaction constants of the production reaction. However, as substrate effects are not the focus of this paper, we will not discuss this any further.

Moiety conservation imposes algebraic constraints at the level of the molecular species. For example the target ‘moiety’ is present in the following forms: free target *t* and its complexes *tc_1_*, *tc_2_, …, tc_n_* with the respective competitors. The concentrations of each of the forms can vary, but their sum remains constant. Similar constraints hold for the competitor ‘moieties’. This leads to the following conservation relationships for the concentrations of the molecular species:
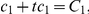


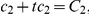



...
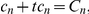


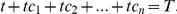



Here the capitals *T* and *C_1_* to *C_n_* are used for total moiety concentrations, while small characters are used for concentrations of monomers and specific complexes.

For this type of networks, we assess the influence of molecular competition for a common target molecule on the fluxes of the competitors. Our approach is based on the theoretical framework of Metabolic Control Analysis and applies to steady states. In the example of Figure 3 *tc_1_* is considered at steady state such that the rates *α_1_* and *β_1_* are equal to each other. This steady-state flux is denoted *J_1_*. Similarly, the flux *J_2_* denotes the steady-state flux through the *tc_2_* pool. We are interested in the sensitivity of the competing fluxes towards the total concentrations of the competitors and the common target. In Metabolic Control Analysis, these sensitivities are expressed by response coefficients. We define an *external* response coefficient of a steady-state system variable [15], [16], *V*, such as a flux, a concentration or any function thereof, to a change in a parameter, *p*, *e.g.* a kinetic parameter or (fixed) external molecular species as:


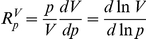
 (see also the Methods section).

This can be understood as the (fractional) change of *V* in response to a small (fractional) change in *p*.

We follow the theory developed in [19]–[21] and therefore also stick to the same choice of symbols as in [20]. Since we are primarily interested in the response to perturbations of the total target or competitor concentrations, *p* will refer to the concentrations of the moieties. Upon a change in such a parameter, the steady state will change and the following *internal* response coefficients can be defined for the response of a system variable *V* to the change in the steady state of a concentration of a species *S_k_* occurring in a moiety conserved cycle.
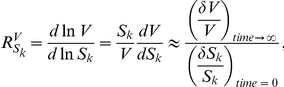
where 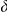 indicates a small change, and time zero is the time at perturbation. In the model of Figure 3 *S_k_* could substitute for any of the molecular species *t*, *c_1_*, *c_2_*, *tc_1_* or *tc_2_*. Sauro [20] showed how the internal response coefficient of a systemic variable *V* (*e.g.* flux) to an initial change in the concentration of a species *S_k_* involved in a conserved sum *T_i_* relates to the external response coefficients towards all the moieties in which this species participates:
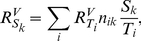
(1)with *n_ik_* the stoichiometry of moiety *k* occurring in cycle *i*. The summation is over all moieties.

Based on the above the following equations for the flux ‘through’ an arbitrary competitor *i* were derived (cf. Text S1 and Text S2).
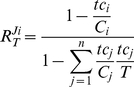
(2)

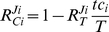
(3)

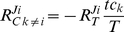
(4)


These are the central equations in our analysis and will give insights into how molecular competition shapes key control and regulatory properties of these networks. Note that each of these response coefficients depends only on the concentrations of conserved moieties of target *T* and specific competitorc *C_i_* and on the concentration of specific target-competitor complexes *tc_i_*. Thus, to evaluate the response coefficients in a real competition network, the underlying rates and rate constants need not be determined.

### Sigma factor competition in bacteria

We will first illustrate the use of equations (2)-(4) for the example of the sigma cycle. Of the seven different types of sigma factors in *E. coli* only σ^70^, σ^54^ and σ^28^ are considered here. They represent by far the three most abundant sigma factors in the exponential phase of *E. coli*.

Maeda *et al.* [10] have reported the following numbers for exponential phase *E. coli* W3110 cells:

Target (RNAP: further shortened to ‘E’): 700 molecules/cell (no free molecules);

Competitor σ^70^: 545 out of 700 molecules/cell are bound to RNAP;

Competitor σ^28^: 100 out of 370 molecules/cell are bound to RNAP;

Competitor σ^54^: 55 out of 110 molecules/cell are bound to RNAP;

Based on this limited information and equations (2)-(4) one calculates the response coefficients of the flux through σ^28^ towards the total concentrations of RNAP and the various sigma factors:
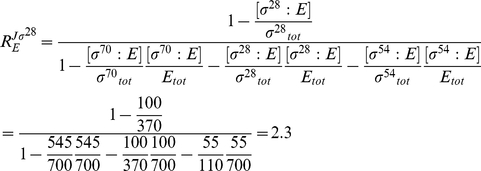


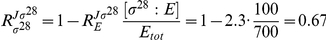


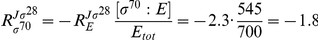


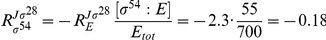



The responses of the fluxes through the other sigma factors are calculated analogously. The full result is presented in Table 1.

**Table 1:** Flux response coefficients 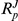 calculated for the example of sigma factor competition.

	RNAP_tot_ #	σ^70^_tot_ #	σ^54^_tot_ #	σ^28^_tot_ #
J_σ70_ (545/700) §	0.70	0.45	−0.055	−0.10
J_σ54_ (55/110) §	1.6	−1.2	0.88	−0.23
J_σ28_ (100/370) §	2.3	−1.8	−0.18	0.67

§ The transcription flux *J* of the gene on which a certain sigma factor acts. The numbers in brackets represent *tc_i_*/*C_i_* for the respective sigma factors.

# The perturbed parameter *p*.

From this table we can already see a number of principles emerging. First, the high sensitivity of the flux of the least saturated sigma factor to an increase in RNAP is remarkable. This follows directly from equation (2). Only the numerators are different for the response coefficients of the different sigma factors to the target and they increase with decreasing *tc_i_/C_i_*. Second, increasing a specific competitor concentration leads to a decrease of any of the other fluxes, *i.e.* all fluxes have a negative response coefficient towards changes in the competing sigma factors. The latter follows from equation (4) if one takes into account that all response coefficients to the target are positive. This competition is stronger, *i.e*. the negative response coefficient towards another competitor becomes larger, when the sigma factor of interest is less saturated with the target. At the same time, the negative response coefficient towards a competitor increases when the latter binds an increasing amount of target. Indeed, comparing the response of the σ^70^ flux to changes in σ^54^ (55/110  =  50% saturation) and σ^28^ (100/370 = 27% saturation), we find that the absolute amount of target bound is determining the competitor strength, and not the saturation. Noteworthy, the strongest competitor has the weakest response to a change in its own concentration.

### Sensitivity to changes in target concentration

Equation (2) gives the response of the *i*-th competitor-binding flux in terms of the amount of the target bound to the different competitors. This is expressed as fractions of the total competitor concentrations *C_i_* occupied by target (*i.e. tc_i_/C_i_*, or in short the ‘competitor saturation’) and the fractions of the total target concentration bound by each competitor (*tc_i_/T*). The value of the numerator equals the fraction of competitor *i* not occupied by target. The denominator has an intuitive symmetrical structure. Based upon this formula, only positive values are possible for these response coefficients (Text S3). Values approach 0 if the numerator approaches 0 and the denominator does not. The first condition is satisfied when most of competitor *i* is present in the bound form. The second condition is satisfied as long as the bulk of the target is not bound to (nearly) saturated competitors.

**Figure 4:**
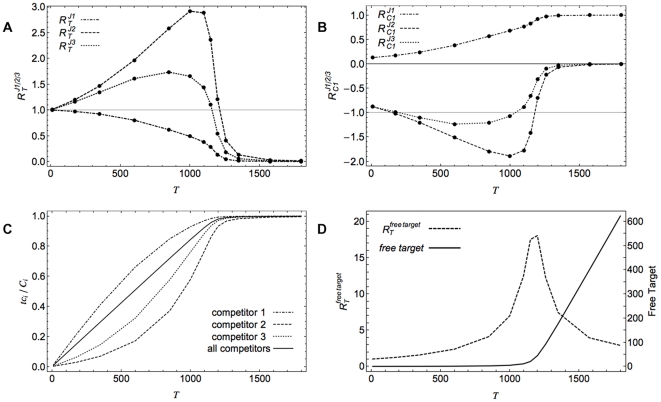
Flux responses in a network of three competitors. Comparison of the flux response coefficients calculated with expressions (2)-(4) (depicted by the big dots), to the values obtained with the basic model (*cf.* Figure 2) for three competitors, simulated at different target levels (10-1800 molecules/cell). The latter data points are represented by the lines. The two approaches give identical results. By analogy with the sigma factor example in the main text the total competitor levels were taken to be 700 molecules/cell for competitor 1 (σ^70^), 370 molecules/cell for competitor 2 (σ^54^) and 110 molecules/cell for competitor 3 (σ^28^). Other parameters needed for simulation were the reaction rate constants: *k_α1,f_* = 24, *k_α2,f_* = 8, *k_α3,f_* = 11 (molecules^−1^.min^−1^), *k_α1,r_* = 3, *k_α2,r_* = 6, *k_α3,r_* = 30 (min^−1^), and *k_β1_’* = *k_β2_’* = *k_β3_’* = 5 (min^−1^). The values were selected to fit the sigma factor example at a total target concentration of 700 molecules/cell (*cf.* Table 1). (A). Flux responses of all individual competitors towards changes in target concentration. (B). Flux response of all individual competitors towards changes in competitor 1. (C). Competitor saturation (*tc_i_*/*C_i_*) as a function of the total target level. The saturation over all competitors is also indicated. (D) The concentration of free target and the response coefficient of the free target concentration to the total target concentration as a function of the total target concentration.

As a general demonstration of the validity of the formula we have calculated the response coefficients for the network from Figure 3, but now with 3 competitors. Simulations were performed for different parameter sets and the response coefficients were calculated (*cf.* Methods). This yielded excellent fits with the corresponding values calculated by equations (2-4). Illustrative examples are shown in Figure 4. Calculating the response coefficients via the more tedious and less intuitive matrix formalism [17] resulted in an exact match with the values from equations (2-4). Even when the perturbations are not infinitesimally small, as expected in many biological settings, our formulas predict the changes in flux quite well (cf. Figure S1).

In Figure 4A, which is inspired by the case of sigma factor competition presented earlier, we find that, at high target concentrations, the flux response coefficients to the total target concentration converge to zero for the fluxes of all three sigma factors. This is due to the fact that all competitors are close to saturation at sufficiently high target concentration and at the same time more and more target is free. In that case the denominator’s value approaches 1, *i.e.* its upper limit, while the numerator approaches 0. Also when at low total target concentration the denominator becomes 1 and the response coefficient becomes nearly equal to the value of the numerator, *i.e.* the fraction of free competitor *i*. This is also shown in Figure 4A: at target concentrations near zero the response coefficient approaches 1.

Interestingly, as illustrated by our examples, response coefficient values higher than 1 are found. This indicates that this process is more sensitive to a change in target concentration than a classical first order reaction or Michaelis-Menten enzyme would be. This phenomenon has been termed ultrasensitivity [22]. For the type of reaction networks investigated here, based on that definition, we have derived a criterion for ultrasensitivity. First we define a saturation fraction ratio, which expresses the relative saturation of competitor *j* w.r.t. competitor *i*:
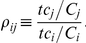



In accordance with equation (2), ultrasensitivity towards the total target concentration *T* is defined by:
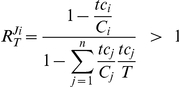



This condition is fulfilled if:
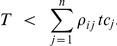



Or, since 
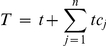
, this condition can be rewritten in terms of the free target concentration *t*:
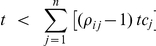



This inequality indicates that ultrasensitivity of flux *i* can only occur if the available free target is below a threshold determined by the degree of saturation of the competitors. This upper bound will increase with an increased binding of the target to the other competitors (higher *tc_j_* values) and/or with an increased fractional occupation of those other competitors (higher *ρ_ij_* values). For both small-scale and large-scale cellular networks it definitely suggests that at least for the least saturated competitors ultrasensitivity to target changes might be a normal phenomenon. Furthermore, since *t* can only be positive, at least one factor *ρ_ij_* has to be higher than 1 for ultrasensitivity to occur. This means that at least one competitor other than *i* must have a higher fractional occupation by the target than *i* itself. In other words, the flux through the strongest binder can never respond ‘ultrasensitively’ to the target concentration. It also follows that ultrasensitivity cannot occur when all competitors are equally strong and therefore have the same degree of saturation. For example, in the case of two competitors, ultrasensitivity of the flux through *tc_1_* occurs when the free target complies with:
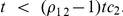



If *ρ_12_*>1 (competitor 2 is more saturated with target than competitor 1) ultrasensitivity to the target is possible for the flux through competitor 1 (however, impossible for the flux through competitor 2). If *ρ_12_*<1 then the opposite is true.

Since the denominator in (2) is the same regardless of which competitor response considered, the ratio between two of these response coefficients equals:
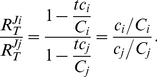
(5)


This means that the relative ratio of the response coefficients of the different competitors to the target is equal to the ratio of the free competitor fractions. The less saturated competitor will always have the highest response to the common resource. This implies that whenever under a particular condition one competitor exhibits ultrasensitivity, then all less saturated competitors will be ultrasensitive too and even more pronounced. See for example Figure 4A, where the response coefficient to the target (RNAP) is higher for the less saturated competitors (σ^54^ and σ^28^ in the example; *cf.* Figure 4C), and this remains the case over the whole range of target concentrations. Above a certain target concentration the ultrasensitivity disappears together with the difference in competitor saturation. At that point the competitors are all near saturation and the free target level increases linearly with the total target level (Figure 4D). This transition is accompanied by a peak in the response coefficient of the free target concentration to total target concentration. At very high target concentrations all response coefficients to the total target concentration will eventually go to zero. Figure S2 shows that this corresponds to a characteristic sigmoidal input-output relation, in terms of total target concentration and reaction flux, respectively (see Figures S3-S5 for related Input/Output plots).

### Sensitivity to changes in total competitor concentrations

Equation (3) describes the response of a competitor flux towards changes in its own total concentration, while equation (4) describes the cross-talk of competitors to competing fluxes.

The response of a competitor flux to a change in its own total concentration is dependent on the response coefficient with respect to the target multiplied with the fraction of *T* bound to that competitor. Based on equations (2) and (3) only values in the interval [0,1] are possible for 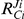 (Text S3). This response coefficient will drop towards zero if 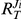 is sufficiently high (typically at low target concentrations) and, at the same time, the bound target represents a significant portion of the total *T* (*cf.* Figure 4B). If total target approaches zero then 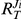 approaches 1 and then 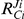 reflects the fraction of total target not bound to competitor *i*. A competitor occurring at much lower levels than the target will per definition always limit its own flux.

The response of the flux of competitor *i* to the total concentration of a competing species *k* is given by equation (4). This equation describes the mutual regulatory influence or crosstalk of the respective competitors. The response of the flux of a competitor *i* towards a change in another competitor *k* equals the response coefficient towards a change in target multiplied by the fraction of total target bound by competitor *k*. According to this equation, only values 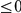 are possible (Text S3), however, in terms of absolute values, ultrasensitivity is indeed possible here. Two parts can be distinguished: the first factor of the expression is a measure of the susceptibility of the flux of competitor *i* to the target, whereas the second factor is specific for competitor *k*. The latter competitor will therefore only have a strong effect if it has bound enough of the target and at the same time the other competitor is sensitive to the target concentration. Intuitively, this makes sense. In absolute values the response coefficient towards a competitor will therefore always be smaller than the response coefficient to the target (compare the first two columns of Table 1; Figure 4). If the total target concentration approaches zero (and 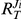 approaches 1) then 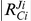 converges to *tc_k_/T*, *i.e.* the fraction of total target bound to competitor *i* (*e.g.* ∼0.8 in Figure 4B). At high target levels the response coefficient towards a competitor will tend to zero just as the response coefficient to the target does. In our example of sigma factor competition high values of RNAP will annihilate all competition and therefore all cross-antagonistic effects.

A further interesting consequence of formula (4) is that for large numbers of (comparably strong) competitors *tc_k_/T* will tend to be small. Therefore the effect of changes in individual competitors will be negligible.

Equation (4) indicates that the flux through *i* can only be ultrasensitive to changes in some competitor *k* if it is ultrasensitive to the target. The concomitant reduction of ultrasensitivity as compared to that towards the target depends on how much of the target is bound to competitor *k*. Re-writing the condition for ultrasensitivity (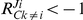, *cf.* Text S4) leads to:
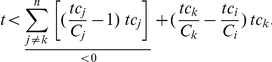



This condition is complex and not immediately insightful. Since the first term is negative, a solution with positive *t* will only exist if the saturation of competitor *k* is higher than that of competitor *i*, and at the same time the total amount of complex *tc_k_* is sufficiently high.

From equations (2) and (4) we can easily derive the following simple relations:
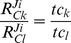
(6)

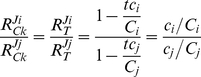
(7)


Equation (6) shows that the relative sensitivity of one flux towards different competitors depends solely on the absolute amount of the target bound to these respective competitors. This is remarkable as one might expect it to correspond to their degree of saturation in accordance with the respective binding strengths of the competitors. In other words, a weaker binder that binds more target because of its higher abundance, will be a stronger competitor. Logically, when the same fractional increase occurs for the two competitors, the one which has bound the most target will again sequester the most target. In the example of Table 1, σ^28^ affects the flux of σ^70^ almost twofold more than σ^54^ does, ‘because’ there are almost twice as many molecules of target bound to σ^28^ than to σ^54^ (100 versus 55).

Equation (7) shows that the relative effect of changing one competitor on the fluxes of the other competitors depends solely on the sensitivity of these fluxes to the common molecular target. This reflects the assumption in the model that the cross-talk between the fluxes of the different competitors is mediated solely by the common target. Furthermore, by using equation (4) it can be shown that the ratio of these responses is equal to the ratio of the free fractions of the ‘influenced’ molecules *i* and *j*. Finally, if one competitor flux exhibits ultrasensitive behavior, then all the less saturated competitor fluxes will do so as well and with higher absolute response coefficients (compare the σ^54^ flux with the σ^28^ flux in Figure 4B).

### Extension and application to translation

Also in protein synthesis competition occurs, with different mRNAs competing for a common set of ribosomes and translation factors. As compared to the reaction scheme of Figure 3, the translation process has two extra complications. First, translation consists of multiple binding and reaction steps instead of one binding and one release step. If we consider transcription and translation as composed of two sequential processes: initiation and elongation (binding and release/production), however, it will still fit to our scheme. Secondly, multiple ribosomes are typically bound and active on one mRNA template, a structure called ‘polysome’. This implies that each mRNA can be in different states depending on the number of ribosomes engaged in protein synthesis. Furthermore, steric interference between ribosomes is then a possibility. To keep the mathematical expressions simple and symmetrical we made the additional assumption that each mRNA binds either its average number of ribosomes (a characteristic polysome size) or nothing, additionally without any steric interference. With this assumption the equations for this case of multiple target binding can be derived in a similar way as for equations (2)-(4) (*cf.* Text S5). The result is quite similar to that of a single target binding, if we make the following replacements in equations (2)-(4).

The fractional occupation (saturation) by target of competitor *i* is replaced by: 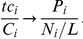



*P_i_* is the average number of ribosomes bound per molecule of mRNA *i*. In an experimental context this typically corresponds to the product of the fraction of mRNA *i* bound to ribosome (‘ribosome occupancy’) and the so-called ‘polysome size’ (the average number of ribosomes in the ribosome-bound fraction of mRNA *i*). *N_i_* is the number of codons of mRNA *i*, and *L* the number of codons occupied by one ribosome.

We perform a similar replacement for the fraction of target bound to competitor *i*:
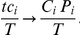



*C_i_* is the total concentration of mRNA *i, T* the total concentration of ribosomes.

Making the necessary substitutions transforms equations (2-4) into:
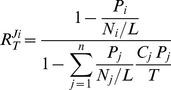
(8)

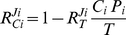
(9)

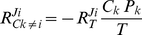
(10)


**Figure 5:**
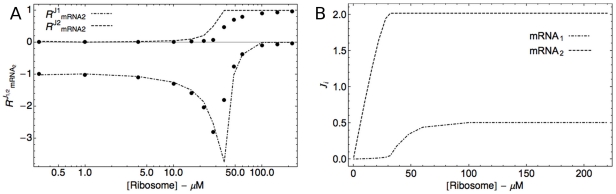
Flux responses in a network of two mRNAs competing for ribosomes. (A). Comparison of the flux response coefficients calculated with expressions (8)-(10) (depicted by big dots), with the values obtained with a model of translation (based on [23]) of two mRNA competitors at different ribosome (target) levels (log-scaled: 0.1–220 µM). The latter data points are represented by the lines. The dashed-dotted line represents the flux response coefficient of the translation of mRNA 1 towards changes in mRNA 2. The dashed line represents the flux response coefficient of the translation of competitor 2 towards changes in competitor 2. The parameters used for the simulation were given the following values: length of ORF = 240 codons, ribosome width  =  12 codons, [competitor 1]  =  0.2 µM, [competitor 2]  =  2 µM. The initiation rate constants were 0.2 (µM.min)^−1^ (competitor 1) and 5 (µM.min)^−1^ (competitor 2), the elongation and termination rate constants were 50 min^−1^ (competitor 1) and 20 min^−1^ (competitor 2). (B). Comparison of the fluxes (in µM.min^−1^) simulated in the same conditions. The dashed-dotted line represents the flux of the translation of mRNA 1, the dashed line represents the flux of the translation of competitor 2.

Since we had to make extra assumptions to derive these equations (2-stage process, no steric interference, all-or-nothing binding), we first compared their outcome to a detailed mathematical model of the translation process in which these assumptions have not been made. In this model translation consists of a single-step initiation phase, a multi-step elongation phase (each codon is represented by a separate state variable) and a single-step termination phase (Figure S6). It extends the model of Heinrich and Rapoport [23], which describes protein synthesis from a single mRNA, to the case of multiple mRNAs competing for ribosome. Importantly, in this model multiple ribosomes can bind each mRNA molecule and, moreover, they can sterically interact. Upon comparing the response coefficients obtained with this detailed model we found the prediction errors to be significant only at very high fractional occupation of mRNA by ribosomes (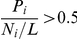). Based on reported large-scale datasets of polysome profiles [24], [25] this is relatively rare in the physiological context. Figure 5B presents the sigmoidal input (total ribosome concentration)-output (reaction flux) relations for such a condition. As depicted in Figure 5A, even for these extreme cases the formula still produces a good approximation.

Analogously to equation (2), equation (8) can be used to derive an (approximate) condition for ultrasensitivity of the translation rate of an individual mRNA to the total ribosome concentration. From equation (8) it can be derived that ultrasensitivity of flux *i* to the ribosome concentration (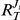
>1) occurs if:
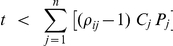



Similarly, the conditions for ultrasensitivity towards individual mRNA species can be derived from the conditions for binding of a single target molecule, by making the same replacements as given above. Analogous to the above it follows that, at low free ribosome concentrations, the least saturated mRNAs will respond ultrasensitively to changes in the total concentration of ribosome. These will also respond ultrasensitively to changes in certain competitors if the latter bind a large fraction of the total ribosome pool (*cf.* equation (4)).

Analogously to equation (7), we find:
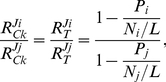
(11)


This implies that the relative sensitivity of two mRNAs to a third competitor is equal to their relative sensitivity to the target concentration, which is determined by their relative saturation by ribosome (the term ‘free’ mRNA is not instructive here).

Noteworthy, one of the most interesting relations is found by adapting equation (6) 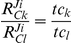 to:
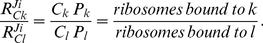
(12)


This equation shows that the relative competitive strength of two mRNAs influencing the flux of a third competitor, is proportional to the relative amount of ribosome bound. Accordingly, the relative ranking according to influence on each ‘other’ mRNA species *i* (‘competitor strength’) is the same across all mRNA species and conditions. Obviously, this independence of *i* is also valid in the case of single target binding.

**Figure 6:**
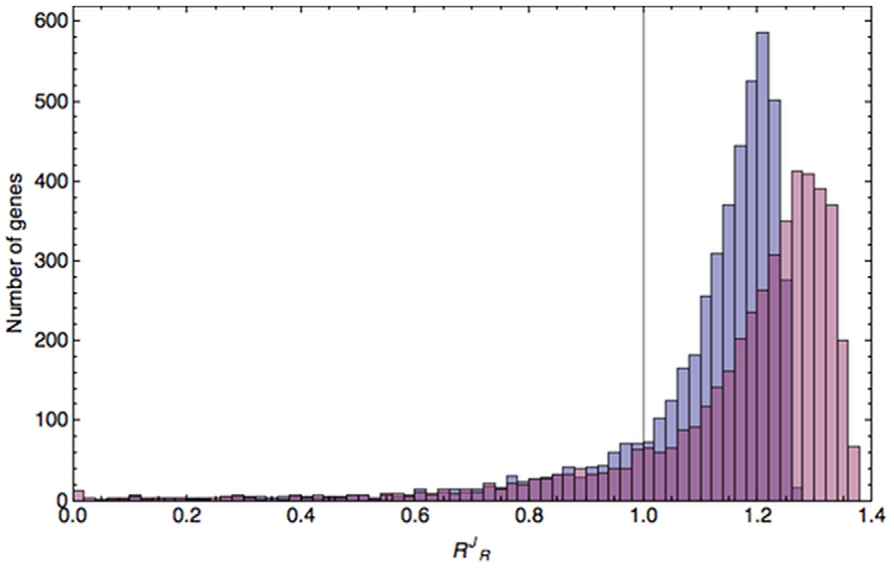
The whole-cell distribution of translation flux response coefficients towards changes in the total ribosome level. The yeast genome-scale datasets published by Siwiak and Zielenkiewicz [26] and by Brockmann *et al.* [27] were used to produce a histogram of the distributions of 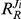
values for two cases. The first distribution (blue bars) was produced by including genome-scale ribosome occupancy values in the calculations; the second (purple bars) was produced assuming ribosome occupancies equal to 1 (more details on the calculations in the Methods section). The vertical line was positioned at 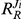 = 1.

Clearly, the power of these equations lies in the possibility to predict the response coefficients for translation of the entire transcriptome, based on the measurable quantities of polysome size (or ribosome density, typically expressed as the number of ribosomes bound per 1000 nucleotides), ORF length, individual mRNA and total ribosome levels. There is no need for any kinetic information. We have applied formula (8) to a dataset consisting of yeast mRNA abundance and ribosome footprints (polysome sizes) as reported by Siwiak and Zielenkiewicz [26], supplemented by ribosome occupancy data from Brockmann *et al.* [27]. In Figure 6 the whole-cell distribution of protein synthesis flux response coefficients towards changes in the total ribosome level is shown for two sets of assumptions. This type of calculation is easily performed with any standard spreadsheet. Strikingly, the response coefficients towards ribosome changes are above 1 for the larger part of the mRNA population. This indicates that translation of the majority of mRNAs has an ultrasensitive response towards changes in cellular ribosome concentration.

**Figure 7:**
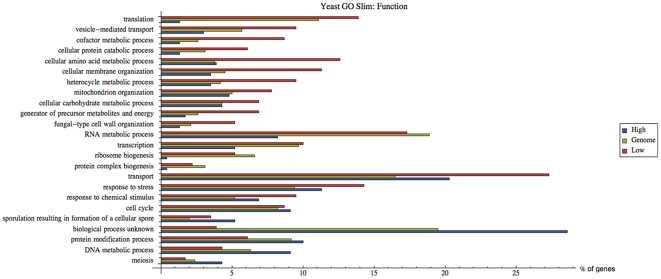
Gene Ontology mapping (in terms of biological process) of high and low response groups. Histogram indicating the relative frequency (as %) of GO classes in high and low response gene sets. The genes corresponding to the mRNAs with the 5% highest (‘High, ’blue bars) or lowest (‘Low’, red bars) response coefficients were pooled and mapped with the GO Slim Mapper tool (http://www.yeastgenome.org/cgi-bin/GO/goSlimMapper.pl), based on the ‘Yeast GO-Slim Process’ GO set. This is a set of high level GO terms that best represent the major biological processes found in *S. cerevisiae*. These terms have been selected by *S. cerevisiae* Genome Database (SGD) curators based on annotation statistics and biological significance. The corresponding percentages for the whole yeast genome (as % of 6310 genes annotated at the moment of analysis, *i.e.* 4-3-2011, in the SGD) are represented by green bars (‘Genome’).

To probe the biological significance of these differences in sensitivities to ribosome changes (and indirectly to mRNA changes), we analyzed the gene composition of the upper and lower 5% bins (231 out of 4621 genes) of the widest distribution (*cf.* Figure 6, colored magenta). The GO Slim Gene Ontology mapper of the ‘*Saccharomyces* Genome Database’ (SGD, http://www.yeastgenome.org/cgi-bin/GO/goSlimMapper.pl) was used to find out how well certain biological categories are represented in the low and high response groups, relative to the *S. cerevisiae* whole genome. Figure 7 depicts the differences based on a set of high level GO terms that represent the major biological processes in *S. cerevisiae* (‘Yeast GO-Slim Process’). Genes related to translation are overrepresented in the low response group, whereas underrepresented in the high response group. That pattern is shared by the vesicle-mediated transport, the cofactor metabolic process, and the cellular protein catabolic process categories. We looked within these biological groups which genes were specifically over- or underrepresented. Within the gene set related to vesicle-mediated transport those necessary for endocytosis are more strongly overrepresented. The other groups have a broader pattern of overrepresentation. For instance, in the translation category we can find more genes related to translation initiation, elongation, regulation, tRNA-amino acylation *etc.* in the low response group. Cellular amino acid metabolic process (specifically amino acylation related genes), cellular membrane organization (again specifically endocytosis related), and heterocycle metabolic process are GO categories with a strong overrepresentation in the low response group, yet without the underrepresentation in the high response group. RNA metabolic process, transcription, ribosome biogenesis, and protein complex biogenesis are specifically strongly underrepresented in the high response group. Less categories show the reverse pattern with underrepresentation in the low response group and overrepresentation in the high response group, with the prominent exception of the category of uncharacterized genes (biological process unknown). The smaller categories protein modification process, DNA metabolic process, and meiosis share this trend. Interestingly, within the latter two categories genes related to DNA repair, DNA recombination, and telomere maintenance are overrepresented. Comparison with an even higher level set of GO terms (‘Super GO-Slim’) resulted in the same broad patterns (Figure S7).

**Figure 8:**
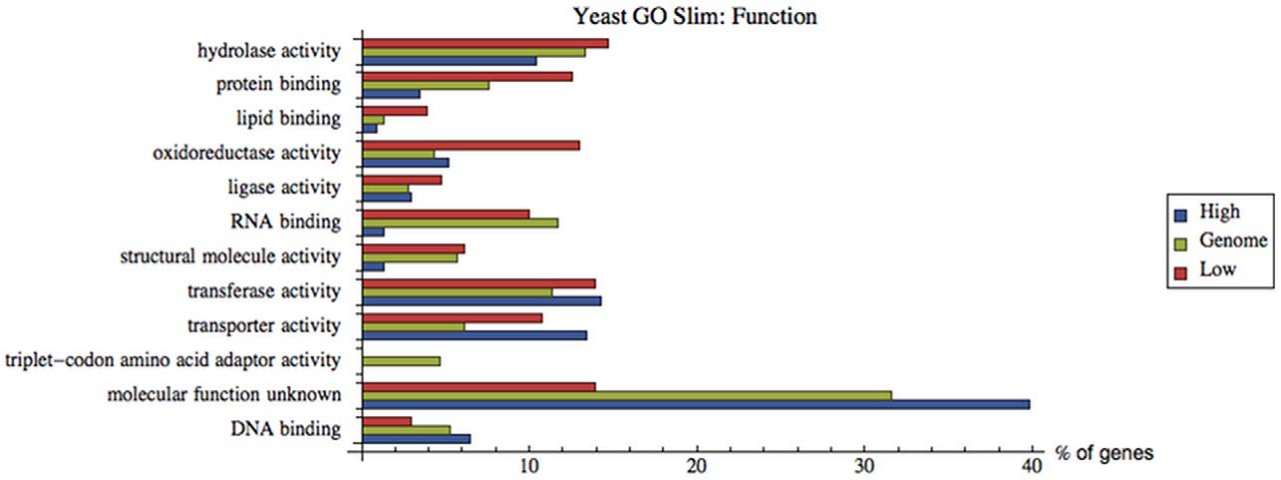
Gene Ontology mapping (in terms of molecular function) of high and low response groups. Histogram indicating the relative frequency (as %) of GO classes in high and low response gene sets. The genes corresponding to the mRNAs with the 5% highest (‘High’, blue bars) or lowest (‘Low’, red bars) response coefficients were pooled and mapped with the GO Slim Mapper tool (http://www.yeastgenome.org/cgi-bin/GO/goSlimMapper.pl), based on the ‘Yeast GO-Slim Function’ GO set. This is a set of high level GO terms that best represent the major biological functions that are found in *S. cerevisiae*. These terms have been selected by *S. cerevisiae* Genome Database (SGD) curators based on annotation statistics and biological significance. The corresponding percentages for the whole yeast genome (as % of 6310 genes annotated at the moment of analysis, *i.e.* 4-3-2011, in the SGD) are represented by green bars (‘Genome’).

Figure 8 depicts the results based on a set of high level GO terms that represent the major molecular functions in *S. cerevisiae* (Yeast Go-Slim Function’). Over- and under-representation for respectively low and high response groups are clear for the protein binding and hydrolase categories. The former category has specifically more genes related to unfolded protein binding and ubiquitin binding. The latter category has specifically more genes with peptidase activity, and with ATPase activity (related to transmembrane movement of substances). Genes with oxidoreductase activity are strongly overrepresented in the low response group. The RNA binding category is strongly underrepresented in the high response group, mainly because of translation related genes. Similar as above, a striking under- and over-representation of unknown molecular function in the low and high response group, respectively, is found. The DNA binding category has the same, albeit less pronounced, pattern.

Finally, we have analyzed the most significant differences based on a set of macromolecular complex terms (‘Cellular Component ontology’: http://www.yeastgenome.org/cgi-bin/GO/goSlimMapper.pl). Figure S8 again shows an overrepresentation of genes involved in ribonucleoprotein complexes in the low-responder group. Interestingly, within this category, telomerase complex related genes are more represented in the high response group.

## Discussion

We have developed a theoretical framework to analyze the properties of networks in which a number of molecular players compete for a common target (in correspondence with the reaction scheme in Figure 3B). These properties are expressed in a precise and unambiguous way by response coefficients. Although the response coefficients are derivatives and quantify the system’s response under small perturbations, we have found that they approximate the flux changes even upon larger perturbations quite well. Normally, the necessity for tedious titration of specific enzyme activities or macromolecule concentrations makes response coefficients experimentally less accessible (for a review of experimental examples *cf.* [28]). The strength of our approach is that the obtained expressions are independent of kinetic parameters and do not require titration of cellular components. Although the system’s responses depend ultimately on the underlying kinetics, they can be inferred solely on the basis of relatively easily obtainable experimental data, such as mRNA levels, and ribosome densities and occupancies in the case of translation. This allows the calculation of response coefficients based on typical genome-scale datasets.

We have studied the response of competing fluxes towards the common target as well as towards the total concentrations of the competitors. In our analysis we focused specifically on conditions that lead to response coefficients substantially different from 1. At relatively high target concentrations, all processes will become insensitive to the target concentration and the response coefficients towards the common target become zero. Conversely, at low target concentration an approximately first order response is predicted. In between, however, response coefficients higher than 1 are possible for all but the most saturated competitor. In accordance with the classical definition [29], [30] we have termed this ultrasensitivity. This corresponds to the differential form of the amplification factor used by Goldbeter and Koshland [18]. In general, a particular competitor will respond more (ultra-) sensitively to its target if the other competitors are closer to saturation, and furthermore if they have bound a relatively large part of the target pool. For ultrasensitivity to competitor changes the conditions are stricter than for ultrasensitivity towards the common target.

Several mechanisms have been reported to produce ultrasensitive behaviour, including cooperativity, multisite phosphorylation, feed-forward loops and enzymes operating under saturation [30]. The latter mechanism is known as ‘zero-order ultrasensitivity’ because a necessary condition is that the modifying and de-modifying enzyme of a covalent modification cycle display zero-order kinetics [22]. Another mechanism accounting for ultrasensitive responses is the ‘branch-point effect’ described by LaPorte and Koshland [29]. This occurs for two (Michaelis-Menten type) enzymes that compete for the same substrate (the ‘branch point’), with one of the enzymes operating near saturation. Kim and Ferrell [31] have demonstrated for mitotic regulation that competition between two sets of phosphorylation sites in Wee1 and between Wee1 and other high- affinity Cdk1 targets can produce ultrasensitive responses. The latter two mechanisms come closest to the competition mechanism which we describe in this paper. Yet, to our knowledge conditions for ultrasensitivity due to competition have never been derived for the type of gene expression networks analysed here.

With respect to regulation, highly saturated competitors are in principle the most robust to changes in cell composition, and the weakly saturated competitors are candidates for highly sensitive regulation. Moreover, the relative response of one flux to different competitors is exactly proportional to the ratio of their target-complex levels. Since the relative ranking of the competitors is the same irrespective of the affected flux, this seems a natural way to describe ‘competitor strength’. Cross-talk between two competitors will obviously be affected by these order relations and therefore will typically be dominated by one competitor.

Due to the condition that the affecting competitor must bind a relatively large portion of the target pool, the antagonistic regulation of competitors is most easily demonstrated with a relatively small number of competitors, such as in the example of sigma factor competition in *E. coli*. Previous studies have reported competition between the housekeeping sigma factor σ^70^ and σ^28^ (σ^F^) [32], σ^38^ (σ^S^) [33], [34], σ^H^ [35] or T4 phage sgp55 [36] respectively. By using our framework, we have attempted to quantitate to what extent this competition exists among endogenous *E. coli* genes. Firstly, we have found that the most abundant and most saturated sigma factor, σ^70^, is the least sensitive to RNAP, followed by σ^54^ (more than twice as sensitive) and σ^28^ (more than three time as sensitive) in accordance with their lower saturation, respectively. The same order is obeyed for the sensitivity to changes in (different) competitors, however; σ^70^ is expected to be hardly sensitive to the others. Indeed, it would make sense during exponential growth for the expression of growth-related and housekeeping genes directed by σ^70^ to be ‘robust’ to changes in accessory sigma factors. Contrary to that, we predict σ^70^ to induce a pronounced negative response on the σ^28^ (flagella and chemotaxis) and σ^54^ (nitrogen and stress responsive) directed genes.

In the stationary phase the predicted cross-talk has been confirmed by overproduction of σ^70^, which mimics the effect of a σ^38^ mutation. Both lead to induction of σ^70^ dependent genes, silencing of σ^38^-dependent genes, and inhibition of stress resistance [8], [33]. Unfortunately, to our knowledge, no measured concentrations of sigma-factor-RNAP complexes are available for the *E. coli* stationary phase. σ^38^ was not included in our calculations since its presence is negligible in the exponential phase [37]. Nevertheless, competition for RNAP would be expected to be even greater during stationary phase because its level decreases to approximately 65% of the log phase level [38]. Furthermore, Jishage [37] showed that, in the same *E. coli* strain W3110, total levels of the main sigma factors remain the same in stationary versus exponential phase, with the exception of σ^38^, which increases to up to 30 percent of that of σ^70^. Interestingly, σ^38^ mutants are highly motile in comparison with wild-type [39], [40], which is attributed to σ^70^ and σ^28^ as these are known to direct flagellar gene transcription. According to our theory it is expected that lowering σ^38^ would affect σ^28^ more than it affects σ^70^.

Partial loss of sigma σ^38^ also results in a growth advantage in stationary phase cultures of *E. coli*, a phenotype termed GASP [41]. In this respect there has been relatively little consideration of the competition between σ^38^ and σ^54^. In fact, the latter competition may be more prevalent than that between σ^38^ and σ^70^ in the context of metabolic functions. Using Phenotype Microarray analysis, it has been found that σ^38^ mutants metabolize 92 different nitrogen sources better than the wild type does, but only 8 carbon sources [40]. Moreover, it has been suggested that the observed growth advantage of *E. coli* σ^38^ mutants versus wild type cells in the mouse colon [42], [43] may at least in part result from better utilization of nitrogen sources due to loss of competition between σ^38^ and σ^54^. Based on our framework and assuming that the relative saturation of σ^70^ and σ^54^ in the stationary phase is similar to that during exponential growth, we can indeed predict that decreasing σ^38^ would alter the transcription flux of σ^54^ directed genes significantly more than that of the σ^70^ directed genes (approximately 2.3 times more for small changes).

In transcription and translation networks, competition is taking place between much larger numbers of competitors. Many different genes (or rather promoters) are competing for RNA polymerase and transcription factors, and thousands of different transcripts are competing for a common set of ribosomes and translation factors. In both cases, it has been shown that the target is only available in limiting concentrations [44],[45] and therefore competition will play a role. To study these networks we adapted the theoretical framework for competitors that bind multiple target molecules. We have demonstrated that even for genome-scale networks it is straightforward to calculate response coefficients from experimental data. Firstly, our results indicate that ultrasensitivity of individual translation fluxes to ribosome changes might be the rule rather than the exception. The long tail at the left side of the resulting distribution histogram (Figure 6) indicates that relatively few mRNAs are insensitive to ribosome changes. In this group we have found a striking overrepresentation of genes (both structurally and functionally) related to translation. Mainly amino-acyl tRNA synthetases and ribosomal proteins, as well as initiation and elongation factors are included. This is further illustrated by the fact that 5 out of the 10 lowest response genes are involved in translation: 2 amino-acyl tRNA synthetases (GUS1 and DPS1), 2 elongation factors (HYP2 and TEF4), and one ribosomal protein (RPP0). Slightly less strongly overrepresented are the genes involved in protein degradation, vesicle-mediated transport (esp. endocytosis), and oxidoreductase activity. This suggests that these processes are robust to changes in ribosome concentration and therefore to changes in other mRNAs. It has been reported that the ribosome concentration varies linearly with the growth rate (*e.g.* [46]). Hence differences in the response coefficients towards the ribosome concentration will determine how the relative rates of translation of different mRNAs co-vary with the growth rate. In particular for translation-related processes this might be important to avoid over-stimulation due to the autocatalytic effect of ribosome synthesis. Remarkably, few GO categories are overrepresented in the group of high-response genes. This might be in part due to drastically higher number of uncharacterized genes in this group. Nevertheless, this group is enriched in genes involved in DNA related processes like DNA recombination and telomerase activity. Although the biological significance of all of these observations remains to be determined (for instance by comparative analysis of different datasets that will become available), one might argue in the latter two cases that these critical activities should indeed be regulatable, rather than robust to changes. Inversely, extra safeguarding mechanisms may be present to protect these both sensitive and crucial genes from the potential harmful consequences of competition.

These large-scale networks typically have hundreds to thousands of competitors that all have a relatively low concentration. Therefore, in contrast to the calculated broad range of target sensitivities, in principle the effect of individual competitor changes on the translation of any of the others will be small since no competitor binds sufficient target. At the same time, the auto-responses are expected to be close to one. Therefore it appears at first sight that the response of large translation networks towards individual mRNAs will be quite simple: the translation of each mRNA will be proportional to its own concentration and hardly sensitive to any of the others. However, this intuition is misleading when groups of competing mRNAs are simultaneously increasing or decreasing. This is not exceptional: the transcription of large groups of genes is co-regulated (the ‘regulon’ concept) and this in turn will affect the abundance of large groups of mRNAs simultaneously (*e.g.* mRNAs encoding ribosomal proteins, developmental transcription programs, *etc*.). Viruses which make use of the gene expression machinery of a host cell provides another example in which competition can be vital ([36]). In conclusion, it is likely that any change of either the ribosome concentration or the overall mRNA composition will result in competition-dependent effects on the individual translation fluxes. Therefore, some translation fluxes can even decrease despite of an increase in the corresponding mRNA levels and vice versa. It follows naturally that the correlation between mRNA levels and protein levels [47], [48] will be affected, without any specific regulatory mechanism that targets individual mRNAs. Evidently, many different factors are at play, for instance molecular stochasticity. Indeed, bursty transcription can lead to noise that is propagated to the protein level [49], [50]. To what extent noise in competitor or target levels gets transmitted to the output fluxes has to be investigated further.

Whereas we have applied the theory to specific examples of transcription and translation, our examples are, however, by no means exhaustive and our theory applies to all levels of the gene expression machinery. One may think *e.g.* about competition of mRNAs for initiation factors or iso-accepting tRNAs that compete for a common amino acid. Moreover, mRNAs compete for the common processing machinery upon transcription, as well as for the common degradation machinery, once they are targeted for degradation. Similarly, the translated proteins will compete not only for a common folding and transport machinery, but also for the common degradation machinery, once they are to be removed [51]–[53]. It is tempting to ascribe all non-linearities in these gene expression processes to dedicated regulation mechanisms. Yet, we have shown that simple competition for a common resource also plays an important role. It remains to be analyzed what the added effect will be of all the non-linearities at different levels of the gene expression cascade.

## Methods

Unless indicated otherwise, all calculations were done in MATHEMATICA. All code is available from the authors upon request.

### Metabolic Control Analysis (MCA)

MCA links ‘global’ control properties of a network to ‘local’ properties (*e.g.* mechanistic details of enzyme-catalyzed reactions; [15], [16]). The local properties are described by elasticity coefficients and are defined by 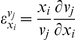. They represent the relative change in a reaction rate *v_i_* as a result of an infinitesimal change in one of its substrate, product, or effector concentrations *x_i_*. Global properties are called response (or sensitivity) coefficients and describe the response of the entire system to small perturbations in parameters, 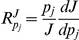. This accounts for the relative change in steady-state flux *J* upon infinitesimal relative change in the value of the parameter *p_j_*. The response coefficient is equally well defined for steady-state metabolite concentration changes as for flux changes. Finally, the control coefficient 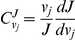, gives the relative change in the steady-state flux *J* in response to an infinitesimal change in one of the reaction rates *v_i_* of the pathway. To compare the response coefficients calculated with equations (2)-(4) to the values obtained with a detailed kinetic model, the following approximate formula was used to calculate response coefficients numerically from the model: 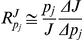. In practice, this method consists of simulating the model in a reference condition and a slightly perturbed condition, *e.g.* a one percent change in parameter *p_j_*. As a control, for a number of cases the more tedious but analytical ‘Matrix Formalism’ [17] was used to verify the exact correspondence (to numerical accuracy of the software) of the results from simulation with the values calculated with equations (2)-(4).

### Models used for validation

The first exemplary model (Figure 4) was inspired by the 3-dimensional sigma factor network described in the Results section. To describe the dynamics of such a network a system of ordinary differential equations was constructed that expresses the mass-balance of the three target-competitor complexes *tc_1_*, *tc_2_* and *tc_3_*. 
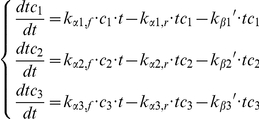



The dynamics of the other time-dependent variables *t*, *c_1_*, *c_2_* and *c_3_* can be directly derived from the system’s state variables using the following mass-conservation relations, which are also used for substituting these variables in the systems right-hand-side.

Total target: 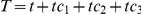


Total competitor ‘1’: 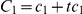


Total competitor ‘2’: 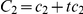


Total competitor ‘3’: 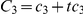


The rate constants were set manually to lie within reasonable bounds and to produce an output roughly in agreement with the values from Table 1. The individual steady-state fluxes *J_i_* were calculated as *k_βI_’ tc_i_*.

The second exemplary model (*cf.* Figure 5), for protein synthesis, is much more complicated since it describes in detail the steric interactions of ribosomes moving along an mRNA template. For a full description we refer to [23]. The only difference with the latter model is that in our case the model was extended to two mRNA templates as opposed to one. Consequently competition could be studied.

Both models were implemented in MATHEMATICA™ v.6 (Wolfram Research, Inc.) and the numerical differential equation solver NDSolve was used for simulation. The steady-state values of the different variables were used to calculate the steady-state fluxes needed for the calculation of the response coefficients (following the procedure described above). Furthermore, the same steady-state concentrations were used as input to equations (2)-(4) and (8)-(10) for comparison.

For the application of expression (8) to a genome-scale data set, we have made use of the data published by Siwiak and Zielenkiewicz [26] since both absolute transcript numbers, ribosome densities and ORF lengths could be easily accessed through the Supplemental Data at the journal website (http://www.ploscompbiol.org). These data were downloaded to MS EXCEL™. As in [26], we assumed a total ribosome concentration of 79 µM. One ribosome was assumed to cover 12 codons (the L-parameter in expressions (8-10); *cf.* [54], [55]). Since 10 data entries had physically impossible ribosome densities, these were set at 8.33, *i.e.* slightly below the maximum value of 8.33… Although the Siwiak and Zielenkiewicz dataset did not contain ribosome occupancies, we have completed it for more than 98 percent with the ribosome occupancy values from the yeast dataset compiled by Brockmann *et al.* [27]. The remaining entries were set to the average ribosome occupancy of the former (*i.e.* 0.75). With some simple algebraic manipulations all 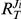
's could be calculated. To demonstrate the importance of including the ribosome occupancies in the calculations, we have re-calculated the 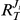
s assuming no unoccupied mRNAs, or therefore all ribosome occupancies equal to 1. Both distributions are depicted in Figure 6. Gene Ontology Mapping was performed with the GO Slim Mapper at the ‘*Saccharomyces cerevisiae* Genome Database’ (SGD project. “Saccharomyces Genome Database” http://www.yeastgSGD project, http://www.yeastgenome.org/, http://www.yeastgenome.org/cgi-bin/GO/goSlimMapper.pl), which maps annotations of a group of genes to more general terms and/or bins them into broad categories, *i.e.* GO Slim terms.

## Supporting Information

Text S1 **Derivation of** **equations (2** **)-(4).**
(DOC)

Text S2 **Proof for specific relations between control coefficients in competing reactions.** Proof for relations between control coefficients in parallel reactions competing for target, needed to derive equation (2)-(4) (*cf.* Text S1).
(DOC)

Text S3 **Short proofs for various properties of response coefficients.**
(DOC)

Text S4
**Ultrasensitivity condition for** 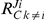 **.** Derivation of ultrasensitivity condition for 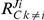.(DOC)

Text S5 **Equations for the general case of multiple target binding.** The formulas for multiple competitors and multiple target binding can be derived analogously to equations (2)-(4) in Text S1.
(DOC)

Figure S1 **Flux responses in a network of three competitors: effect of large perturbations.** Comparison of the flux response coefficients (towards changes in target concentration) calculated with expression (2) (depicted by the big dots), to the values obtained with the basic model (*cf.* Figure 3) for three competitors, simulated at different target levels (10-1800 molecules/cell). These response coefficients were derived from model simulations at different relative perturbations of the total target level: 1% (black lines), 5% (red lines), 10% (green lines), and 15% (purple lines), respectively. The two approaches give nearly identical results for the 1% perturbation and the difference increases gradually with larger perturbations. By analogy with the sigma factor example in the main text the total competitor levels were taken to be 700 molecules/cell for competitor 1 (σ^70^), 370 molecules/cell for competitor 2 (σ^54^) and 110 molecules/cell for competitor 3 (σ^28^). Other parameters needed for simulation are as in Figure 4A.
(TIF)

Figure S2 **Input-output relations in a network of three competitors.** Comparison of the fluxes (in molecules.min^−1^) obtained through simulation of the basic model (*cf.* Figure 3) for three competitors, simulated at different target levels (10-1800 molecules/cell). By analogy with the sigma factor example in the main text the total competitor levels were taken to be 700 molecules/cell for competitor 1 (σ^70^), 370 molecules/cell for competitor 2 (σ^54^) and 110 molecules/cell for competitor 3 (σ^28^). Other parameters needed for simulation were the reaction rate constants: *k_α1,f_*  = 24, *k_α2,f_*  = 8, *k_α3,f_*  = 11 (molecules^−1^.min^−1^), *k_α1,r_*  = 3, *k_α2,r_* = 6, *k_α3,r_* = 30 (min^−1^), and *k_β1_’* = *k_β2_’* = *k_β3_’* = 5 (min^−1^). The values were selected to fit the sigma factor example at a total target concentration of 700 molecules/cell (*cf.* Table 1).
(TIF)

Figure S3 **Input-output relations in a network of three competitors.** (A). Flux response coefficients calculated for three competitors, simulated at different levels (1-3000 molecules/cell) of competitor 1. By analogy with the sigma factor example in the main text the total competitor levels that were fixed, were taken to be 370 molecules/cell for competitor 2 (σ^54^) and 110 molecules/cell for competitor 3 (σ^28^). The total target level was set at 700 molecules/cell. Other parameters needed for simulation were as in Figure S2. (B). Plot of input (total competitor level)/output (competitor flux in molecules.min^−1^) relation corresponding to (A).
(TIF)

Figure S4 **Input-output relations in a network of three competitors.** (A). Flux response coefficients calculated for three competitors, simulated at different levels (1-3000 molecules/cell) of competitor 2. By analogy with the sigma factor example in the main text the total competitor levels that were fixed, were taken to be 700 molecules/cell for competitor 1 (σ^70^) and 110 molecules/cell for competitor 3 (σ^28^). The total target level was set at 700 molecules/cell. Other parameters needed for simulation were as in Figure S2. (B). Plot of input (total competitor level)/output (competitor flux in molecules.min^−1^) relation corresponding to (A).
(TIF)

Figure S5 **Input-output relations in a network of three competitors.** (A). Flux response coefficients calculated for three competitors, simulated at different levels (1-3000 molecules/cell) of competitor 3. By analogy with the sigma factor example in the main text the total competitor levels that were fixed, were taken to be 700 molecules/cell for competitor 1 (σ^70^) and 370 molecules/cell for competitor 2 (σ^54^). The total target level was set at 700 molecules/cell. Other parameters needed for simulation were as in Figure S2. (B). Plot of input (total competitor level)/output (competitor flux in molecules.min^−1^) relation corresponding to (A).
(TIF)

Figure S6 **Translation model.** The validity of expressions (8-10) was tested by comparison with a protein synthesis model based on Heinrich and Rapoport’s model [23]. This model takes into account the steric interactions of ribosomes as hard bodies occupying a fixed number of codons, moving along an mRNA template. Each state variable describes the (fractional) occupation by the front of a ribosome of an individual codon (the number of state variables is equal to the number of codons). Translation initiation is represented as an irreversible bimolecular reaction proportional to the available (free) ribosomes (subunit dissociation is not taken into account) and the fraction of free start sites (i.e. at the AUG codon with no sterically interacting ribosomes nearby). The translation elongation at a specific codon is represented by an irreversible unimolecular reaction proportional to the (fractional) occupation at that codon by the front of a ribosome. The corresponding rate constant is weighted by probability that the next codon is free to be occupied given that specific codon is occupied. The translation termination reaction is represented as an irreversible unimolecular reaction proportional to the (fractional) occupation of the last codon. To allow us to study competition the original model was extended to two mRNA templates as opposed to one. This means that three conservation relations exist i.e. for the total ribosome level and the total levels of the individual mRNAs.
(TIF)

Figure S7 **Gene Ontology mapping (in terms of high level biological process) of high and low response groups.** Histogram indicating the relative frequency (as %) of GO classes in high and low response gene sets. The genes corresponding to mRNAs with the 5% highest (‘High’, blue bars) or lowest (‘Low’, red bars) response coefficients were pooled and mapped with the GO Slim Mapper tool (http://www.yeastgenome.org/cgi-bin/GO/goSlimMapper.pl), based on the ‘Super GO-Slim Process’ GO set. This is a small set of very broad, high level GO Biological Process terms, useful for binning groups of genes in general categories. The corresponding percentages for the whole yeast genome (as % of 6310 genes annotated at the moment of analysis, *i.e.* 4-3-2011, in the SGD) are represented by green bars (‘Genome’).
(TIF)

Figure S8 **Gene Ontology mapping (in terms of molecular complex) of high and low response groups.** Histogram indicating the relative frequency (as %) of GO classes in high and low response gene sets. The genes corresponding to the mRNAs with the 5% highest (‘High’, blue bars) and lowest (‘Low’, red bars) response coefficients were pooled and mapped with the GO Slim Mapper tool (http://www.yeastgenome.org/cgi-bin/GO/goSlimMapper.pl), based on the ‘Cellular Component’ ontology. This is a set of granular protein complex terms, useful for determining whether your protein of interest is a member of a particular complex. The corresponding percentages for the whole yeast genome (as % of 6310 genes annotated at the moment of analysis, *i.e.* 4-3-2011, in the SGD) are represented by green bars (‘Genome’).
(TIF)
