# Supplementary material for: How Molecular Competition Influences Fluxes in Gene Expression Networks
Source: PLoS One. 2011 Dec 5;6(12):e28494. doi: 10.1371/journal.pone.0028494 (PMC3230629; doi:10.1371/journal.pone.0028494)
Supplement: Text S1 — Derivation of equations (2 )-(4). (DOC) [file pone.0028494.s001.doc]

The simplified model illustrated in Figure 3 with 2 competitors *c_1_* and *c_2_*, competing for a ‘target’ *t*, is easily extended to *n* competitors. In the binding steps (with rates *α_i_*) the competitors form a complex with the target. In the release steps (with rates *β_i_*) product (*p_i_*) is generated and competitors and target are released.

The following expression for the internal response coefficient is used:

$R_{S_{k}}^{V}=\sum_{i} R_{T_{i}}^{V}n_{ik}\frac{S_{k}}{T_{i}}$ , (1)

where *n_ik_* is the stoichiometric coefficient of moiety *k* occuring in cycle *i*.

Conservation relations:

$t+tc_{1}+tc_{2}+...+tc_{n}=T$,

$c_{1}+tc_{1}=C_{1}$,

$c_{2}+tc_{2}=C_{2}$,

…

$c_{n}+tc_{n}=C_{n}$.

Applying equation (1) gives for the response of the steady-state flux *J_i_* of competitor *i* to changes in the various internal variables:

$R_{t}^{Ji}=R_{T}^{Ji}\frac{t}{T}$,

$R_{c1}^{Ji}=R_{C1}^{Ji}\frac{c_{1}}{C_{1}}$,

$R_{c2}^{Ji}=R_{C2}^{Ji}\frac{c_{2}}{C_{2}}$,

…

$R_{cn}^{Ji}=R_{Cn}^{Ji}\frac{c_{n}}{C_{n}}$.

$R_{tc1}^{Ji}=R_{T}^{Ji}\frac{tc_{1}}{T}+R_{C1}^{Ji}\frac{tc_{1}}{C_{1}}$,

$R_{tc2}^{Ji}=R_{T}^{Ji}\frac{tc_{2}}{T}+R_{C2}^{Ji}\frac{tc_{2}}{C_{2}}$,

…

$R_{tcn}^{Ji}=R_{T}^{Ji}\frac{tc_{n}}{T}+R_{Cn}^{Ji}\frac{tc_{n}}{C_{n}}$.

Then we calculate the following sum:

$R_{t}^{Ji}+R_{tc1}^{Ji}+R_{tc2}^{Ji}+...+R_{tcn}^{Ji}=R_{T}^{Ji}\frac{t}{T}+R_{T}^{Ji}\frac{tc_{1}}{T}+R_{C1}^{Ji}\frac{tc_{1}}{C_{1}}+R_{T}^{Ji}\frac{tc_{2}}{T}+R_{C2}^{Ji}\frac{tc_{2}}{C_{2}}+...+R_{T}^{Ji}\frac{tc_{n}}{T}+R_{Cn}^{Ji}\frac{tc_{n}}{C_{n}}$,

$=R_{T}^{Ji}\left(\frac{t}{T}+\frac{tc_{1}}{T}+\frac{tc_{2}}{T}+...+\frac{tc_{n}}{T}\right)+R_{C1}^{Ji}\frac{tc_{1}}{C_{1}}+R_{C2}^{Ji}\frac{tc_{2}}{C_{2}}+...+R_{Cn}^{Ji}\frac{tc_{n}}{C_{n}}$,

$=R_{T}^{Ji}+R_{C1}^{Ji}\frac{tc_{1}}{C_{1}}+R_{C2}^{Ji}\frac{tc_{2}}{C_{2}}+...+R_{Cn}^{Ji}\frac{tc_{n}}{C_{n}}$ (13)

To rewrite these equations we will use the generalized flux connectivity theorem (equation 19 in [20]):

$R_{S_{k}}^{J}=\sum_{i=1}^{n} C_{v_{i}}^{J}\varepsilon_{S_{k}}^{v_{i}}$,

which sums over all rates *v_i_* that are directly affected by a change in the internal metabolite *S_k_*.

Assuming furthermore that the rates depend proportionally on their respective substrate concentrations (mass-action kinetics, all elasticity coefficients *ε* towards a substrate of the reaction are 1) the following is derived:

$R_{t}^{Ji}=C_{\alpha1}^{Ji}\varepsilon_{t}^{\alpha1}+C_{\alpha2}^{Ji}\varepsilon_{t}^{\alpha2}+...+C_{\alpha n}^{Ji}\varepsilon_{t}^{\alpha n}$,

$R_{c1}^{Ji}=C_{\alpha1}^{Ji}\varepsilon_{c1}^{\alpha1}$,

$R_{c2}^{Ji}=C_{\alpha2}^{Ji}\varepsilon_{c2}^{\alpha2}$,

…

$R_{cn}^{Ji}=C_{\alpha n}^{Ji}\varepsilon_{cn}^{\alpha n}$.

$R_{tc1}^{Ji}=C_{\beta1}^{Ji}\varepsilon_{tc1}^{\beta1}$,

$R_{tc2}^{Ji}=C_{\beta2}^{Ji}\varepsilon_{tc2}^{\beta2}$,

…

$$R_{tcn}^{Ji}=C_{\beta n}^{Ji}\varepsilon_{tcn}^{\beta n}$$

Then we calculate the following sum:

$\begin{matrix} R_{t}^{Ji}+R_{tc1}^{Ji}+R_{tc2}^{Ji}+...+R_{tcn}^{Ji}=C_{\alpha1}^{Ji}\left(\varepsilon_{t}^{\alpha1}+\varepsilon_{tc1}^{\alpha1}\right)+C_{\alpha2}^{Ji}\left(\varepsilon_{t}^{\alpha2}+\varepsilon_{tc2}^{\alpha2}\right)+...+C_{\alpha n}^{Ji}\left(\varepsilon_{t}^{\alpha n}+\varepsilon_{tcn}^{\alpha n}\right) \\ \\ +C_{\beta1}^{Ji}\varepsilon_{tc1}^{\beta1}+C_{\beta2}^{Ji}\varepsilon_{tc2}^{\beta2}+...+C_{\beta n}^{Ji}\varepsilon_{tcn}^{\beta n} \end{matrix}$,

The elasticities can then be calculated based on the proposed rate equations (Figure 3B). The rate equations for competitor *i* read:

$\alpha_{i}=k_{\alpha i,f}\cdot c_{i}\cdot t-k_{\alpha i,r}\cdot tc_{i}$,

$\beta_{i}=k'_{\beta i}\cdot tc_{i}$.

With the elasticity defined as $\varepsilon_{x_{i}}^{v_{j}}=\frac{x_{i}}{v_{j}}\frac{\partial v_{j}}{\partial x_{i}}$ (*cf.* Methods), we can derive:

$\varepsilon_{t}^{\alpha i}+\varepsilon_{tci}^{\alpha i}=\frac{t}{\alpha_{i}}k_{\alpha i,f} c_{i}+\frac{tc_{i}}{\alpha_{i}}\left(-k_{\alpha i,r}\right)=1$,

$\varepsilon_{tci}^{\beta i}=\frac{tc_{i}}{\beta_{i}}k'_{\beta i}=1$.

Therefore:

$R_{t}^{Ji}+R_{tc1}^{Ji}+R_{tc2}^{Ji}+...+R_{tcn}^{Ji}=C_{\alpha1}^{Ji}+C_{\alpha2}^{Ji}+...+C_{\alpha n}^{Ji}+C_{\beta1}^{Ji}+C_{\beta2}^{Ji}+...+C_{\beta n}^{Ji}\equiv1$ (14)

This relationship equates to 1 according to the summation theorem of metabolic control analysis.

Then equation (13) and (14) lead to an expression for the response coefficient for flux *J_i_* with respect to the target concentration in terms of the response coefficients of this flux to the total competitor levels and their bound fractions:

$R_{T}^{Ji}=1-R_{C1}^{Ji}\frac{tc_{1}}{C_{1}}-R_{C2}^{Ji}\frac{tc_{2}}{C_{2}}-...-R_{Cn}^{Ji}\frac{tc_{n}}{C_{n}}$. (15)

A similar procedure for the following sum:

$R_{ci}^{Ji}+R_{tci}^{Ji}$,

with

$C_{\alpha i}^{Ji}+C_{\beta i}^{Ji}\equiv1$ (proof in Text S1),

leads to:

$R_{Ci}^{Ji}=1-R_{T}^{Ji}\frac{tc_{i}}{T}$, (3)

which is an expression for the response coefficient for flux *J_i_* with respect to competitor *i* in terms of the response coefficient of this flux to the total target level and the fraction of total target bound to that competitor.

And for the following sum: $R_{ck}^{Ji}+R_{tck}^{Ji}$ (*i≠k*)

with $C_{\alpha k}^{Ji}+C_{\beta k}^{Ji}\equiv0$ (proof in Text S1)

leads to:

$R_{C k\neq i}^{Ji}=-R_{T}^{Ji}\frac{tc_{k}}{T}$, (4)

which is an expression for the response coefficient for flux *J_i_* with respect to competitor *k* in terms of the response coefficient of this flux to the total target level and the fraction of total target bound to competitor *k*.

Using the expressions (3) and (4), we can then solve equation (15) for the response coefficient of the flux to the total concentration of target:

$R_{T}^{Ji}=\frac{1-\frac{tc_{i}}{C_{i}}}{1-\sum_{j}^{n} \frac{tc_{j}}{C_{j}}\frac{tc_{j}}{T}}$ (2)
