# Supplementary material for: How Molecular Competition Influences Fluxes in Gene Expression Networks
Source: PLoS One. 2011 Dec 5;6(12):e28494. doi: 10.1371/journal.pone.0028494 (PMC3230629; doi:10.1371/journal.pone.0028494)
Supplement: Text S2 — Proof for specific relations between control coefficients in competing reactions. Proof for relations between control coefficients in parallel reactions competing for target, needed to derive equation (2)-(4) (cf. Text S1). (DOC) [file pone.0028494.s002.doc]

According to the summation theorem for flux-control coefficients in matrix form [17]:

$C^{J}K = K$. (16)

*I.e.* the product of the matrix of flux-control coefficients *C^J^* and the kernel (or null-space) *K* of the stoichiometric matrix equals the *K*-matrix.

Starting from the stoichiometric matrix *N* (corresponding to the general scheme with n competitors and single target binding; *cf.* Figure 3A), with the variables (*t*, *c_1_*, …)represented by the rows and the reaction rates (*α_1_*, *β_1_*, …) represented by the columns:

$$\begin{matrix} & \alpha_{1} & \beta_{1} & \alpha_{2} & \beta_{2} & \ldots& \alpha_{n} & \beta_{n} \end{matrix}$$

*N* = $\begin{matrix} t \\ c_{1} \\ tc_{1} \\ c_{2} \\ tc_{2} \\ \vdots\\ c_{n} \\ tc_{n} \end{matrix}$ $\left[\begin{matrix} -1 & 1 & -1 & 1 & \cdots& -1 & 1 \\ -1 & 1 & 0 & 0 & \cdots& 0 & 0 \\ 1 & -1 & 0 & 0 & \cdots& 0 & 0 \\ 0 & 0 & -1 & 1 & \cdots& 0 & 0 \\ 0 & 0 & 1 & -1 & \cdots& 0 & 0 \\ \vdots& \vdots& \vdots& \vdots& \ddots& \vdots& \vdots\\ 0 & 0 & 0 & 0 & \cdots& -1 & 1 \\ 0 & 0 & 0 & 0 & \cdots& 1 & -1 \end{matrix}\right]$

Row-reduction leads to:

$K=\left[\begin{matrix} 1 & 0 & \ldots& 0 \\ 1 & 0 & \cdots& 0 \\ 0 & 1 & \cdots& 0 \\ 0 & 1 & \cdots& 0 \\ \vdots& \vdots& \ddots& \vdots\\ 0 & 0 & \cdots& 1 \\ 0 & 0 & \cdots& 1 \end{matrix}\right]$,

Substitution into equation (16) results in:

$\left[\begin{matrix} C_{\alpha1}^{J\alpha1} & C_{\beta1}^{J\alpha1} & C_{a,2}^{J\alpha1} & C_{\beta2}^{J\alpha1} & \cdots& C_{a,n}^{J\alpha1} & C_{\beta n}^{J\alpha1} \\ C_{\alpha1}^{J\beta1} & C_{\beta1}^{J\beta1} & C_{\alpha2}^{J\beta1} & C_{\beta2}^{J\beta1} & \cdots& C_{\alpha n}^{J\beta1} & C_{\beta n}^{J\beta1} \\ C_{\alpha1}^{J\alpha2} & C_{\beta1}^{J\alpha2} & C_{\alpha2}^{J\alpha2} & C_{\beta2}^{J\alpha2} & \cdots& C_{\alpha n}^{J\alpha2} & C_{\beta n}^{J\alpha2} \\ C_{\alpha1}^{J\beta2} & C_{\beta1}^{J\beta2} & C_{\alpha2}^{J\beta2} & C_{\beta2}^{J\beta2} & \cdots& C_{\alpha n}^{J\beta2} & C_{\beta n}^{J\beta2} \\ \vdots& \vdots& \vdots& \vdots& \ddots& \vdots& \vdots\\ C_{\alpha1}^{J\alpha n} & C_{\beta1}^{J\alpha n} & C_{\alpha2}^{J\alpha n} & C_{\beta2}^{J\alpha n} & \cdots& C_{\alpha n}^{J\alpha n} & C_{\beta n}^{J\alpha n} \\ C_{\alpha1}^{J\beta n} & C_{\beta1}^{J\beta n} & C_{\alpha2}^{J\beta n} & C_{\beta2}^{J\beta n} & \cdots& C_{\alpha n}^{J\beta n} & C_{\beta n}^{J\beta n} \end{matrix}\right]$ . $\left[\begin{matrix} 1 & 0 & \ldots& 0 \\ 1 & 0 & \cdots& 0 \\ 0 & 1 & \cdots& 0 \\ 0 & 1 & \cdots& 0 \\ \vdots& \vdots& \ddots& \vdots\\ 0 & 0 & \cdots& 1 \\ 0 & 0 & \cdots& 1 \end{matrix}\right]$ = $\left[\begin{matrix} 1 & 0 & \ldots& 0 \\ 1 & 0 & \cdots& 0 \\ 0 & 1 & \cdots& 0 \\ 0 & 1 & \cdots& 0 \\ \vdots& \vdots& \ddots& \vdots\\ 0 & 0 & \cdots& 1 \\ 0 & 0 & \cdots& 1 \end{matrix}\right]$

Or:

$$\begin{matrix} C_{\alpha1}^{J\alpha1} + C_{\beta1}^{J\alpha1} = 1 \\ C_{\alpha1}^{J\beta1} + C_{\beta1}^{J\beta1} = 1 \\ C_{\alpha1}^{J\alpha2} + C_{\beta1}^{J\alpha2} = 0 \\ C_{\alpha1}^{J\beta2} + C_{\beta1}^{J\beta2} = 0 \\ \ldots\\ C_{\alpha1}^{J\alpha n} + C_{\beta1}^{J\alpha n} = 0 \\ C_{\alpha1}^{J\beta n} + C_{\beta1}^{J\beta n} = 0 \\ \ldots\end{matrix}$$

Since the fluxes for reactions *α* and *β* of the same competitor are equal in the steady-state this can be written as:

$$C_{\alpha j}^{Ji} + C_{\beta j}^{Ji} = \left\{\begin{matrix} 1 & i=j \\ 0 & i\neq j \end{matrix}\right.$$

(or: $C_{\alpha j}^{Ji} + C_{\beta j}^{Ji} = \delta_{ij}$)

Ordinarily, we are working with normalized response and control coefficients. In that case the matrices concerned have to be scaled:

$\bar{C}^{J}= (D^{J})^{-1} C^{J}D^{J}$ and

$\bar{K} = (D^{J})^{-1} K D^{J}$ ,

with the diagonal matrix $D^{J}$, which has the steady-state fluxes as diagonal elements, and $\bar{C}^{J}$ and $\bar{K}$ the scaled forms of $C^{J}$and $K$, respectively. The resulting relationships between the control coefficients, however, are the same.


