# Supplementary material for: How Molecular Competition Influences Fluxes in Gene Expression Networks
Source: PLoS One. 2011 Dec 5;6(12):e28494. doi: 10.1371/journal.pone.0028494 (PMC3230629; doi:10.1371/journal.pone.0028494)
Supplement: Text S3 — Short proofs for various properties of response coefficients. (DOC) [file pone.0028494.s003.doc]

*$R_{T}^{Ji}\in\left[0,+\infty\right)$

Assume that $R_{T}^{Ji}=\frac{1-\frac{tc_{i}}{C_{i}}}{1-\sum_{j}^{n} \frac{tc_{j}}{C_{j}}\frac{tc_{j}}{T}}<0$,

then either $1-\frac{tc_{i}}{C_{i}}<0$and thereby $\frac{tc_{i}}{C_{i}}>1$ which is impossible,

or $1-\sum_{j}^{n} \frac{tc_{j}}{C_{j}}\frac{tc_{j}}{T}<0\Leftrightarrow1<\sum_{j}^{n} \frac{tc_{j}}{C_{j}}\frac{tc_{j}}{T} < \sum_{j}^{n} \frac{tc_{j}}{C_{j}}<1$which is a contradiction.



*$R_{Ci}^{Ji}\in\left[0,1\right]$

Assume that $R_{Ci}^{Ji}=1-R_{T}^{Ji}\frac{tc_{i}}{T}>1$,

then: $R_{T}^{Ji}\frac{tc_{i}}{T}<0$, which is impossible since $R_{T}^{Ji}\geq0$ and $\frac{tc_{i}}{T}>0$.



Assume that $R_{Ci}^{Ji}=1-R_{T}^{Ji}\frac{tc_{i}}{T}<0$,

then: $R_{T}^{Ji}\frac{tc_{i}}{T}>1$.

Substituting with equation (7) yields: $\frac{\frac{tc_{i}}{T}-\frac{tc_{i}}{C_{i}}\frac{tc_{i}}{T}}{1-\sum_{j}^{n} \frac{tc_{j}}{C_{j}}\frac{tc_{j}}{T}}>1$.

Rearranging yields: $tc_{i}+\sum_{j\neq i}^{n} \frac{tc_{j}}{C_{j}}tc_{j}>T$.

Replacing *T*: $tc_{i}+\sum_{j\neq i}^{n} \frac{tc_{j}}{C_{j}}tc_{j}>t+\sum_{j}^{n} tc_{j}$,

gives:

$t+\sum_{j\neq i}^{n} tc_{j}(\mathop{\underline{1-\frac{tc_{j}}{C_{j}}})}\limits_{\geq0} <0$, which is impossible since all entities on the left are $\geq$ 0.



*$R_{C k\neq i}^{Ji}\in\left(-\infty,0\right]$

Assume$R_{C k\neq i}^{Ji}=-R_{T}^{Ji}\frac{tc_{k}}{T}>0$

Then: $R_{T}^{Ji}\frac{tc_{k}}{T}<0$, which is impossible since all entities on the left are $\geq$ 0.


