# Supplementary material for: How Molecular Competition Influences Fluxes in Gene Expression Networks
Source: PLoS One. 2011 Dec 5;6(12):e28494. doi: 10.1371/journal.pone.0028494 (PMC3230629; doi:10.1371/journal.pone.0028494)
Supplement: Text S4 — Ultrasensitivity condition for . Derivation of ultrasensitivity condition for . (DOC) [file pone.0028494.s004.doc]

Ultrasensitivity condition for $R_{C k\neq i}^{Ji}$:

$R_{C k\neq i}^{Ji}<-1$.

Substituting for $R_{C k\neq i}^{Ji}$:

$-\frac{1-\frac{tc_{i}}{C_{i}}}{1-\sum_{j}^{n} \frac{tc_{j}}{C_{j}}\frac{tc_{j}}{T}}\cdot\frac{tc_{k}}{T}<-1$.

Re-arranging:

$\frac{tc_{k}}{T}-\frac{tc_{i}}{C_{i}}\frac{tc_{k}}{T}+\sum_{j}^{n} \frac{tc_{j}}{C_{j}}\frac{tc_{j}}{T}>1$.

Re-arranging and substituting *T*:

$tc_{k}-\frac{tc_{i}}{C_{i}}tc_{k}+\sum_{j}^{n} \frac{tc_{j}}{C_{j}}tc_{j}>t+\sum_{j}^{n} tc_{j}$,

$t<\mathop{\underline{\sum_{j\neq k}^{n} \left[(\frac{tc_{j}}{C_{j}}-1) tc_{j}\right]}}\limits_{<0} +(\frac{tc_{k}}{C_{k}}-\frac{tc_{i}}{C_{i}}) tc_{k}$.

Since the first part at the right-hand-side consists of negative terms the last term has to be more positive. This will only occur if the saturation of competitor *k* is higher than that of competitor *i*, and at the same time the total amount of *tc_k_* complex is sufficiently high.
