# Supplementary material for: How Molecular Competition Influences Fluxes in Gene Expression Networks
Source: PLoS One. 2011 Dec 5;6(12):e28494. doi: 10.1371/journal.pone.0028494 (PMC3230629; doi:10.1371/journal.pone.0028494)
Supplement: Text S5 — Equations for the general case of multiple target binding. The formulas for multiple competitors and multiple target binding can be derived analogously to equations (2)-(4) in Text S1. (DOC) [file pone.0028494.s005.doc]

The formulas for multiple competitors and multiple target binding can be derived analogously to Text S1. With *n_i_*, *n_j_* and *n_k_* designating the number of target molecules bound to the respective complexes *tc_i_*, *tc_j_* and *tc_k_*, we get:

$R_{T}^{Ji}=\frac{1-\frac{tc_{i}}{C_{i}}}{1-\sum_{j}^{n} n_{j}\frac{tc_{j}}{C_{j}}\frac{tc_{j}}{T}}$, (17)

$R_{Ci}^{Ji}=1-R_{T}^{Ji}n_{i}\frac{tc_{i}}{T}$, (18)

$R_{C k\neq i}^{Ji}=-R_{T}^{Ji}n_{k}\frac{tc_{k}}{T}$. (19)
