# Supplementary figures and images for: How Molecular Competition Influences Fluxes in Gene Expression Networks
Source: PLoS One. 2011 Dec 5;6(12):e28494. doi: 10.1371/journal.pone.0028494 (PMC3230629; doi:10.1371/journal.pone.0028494)

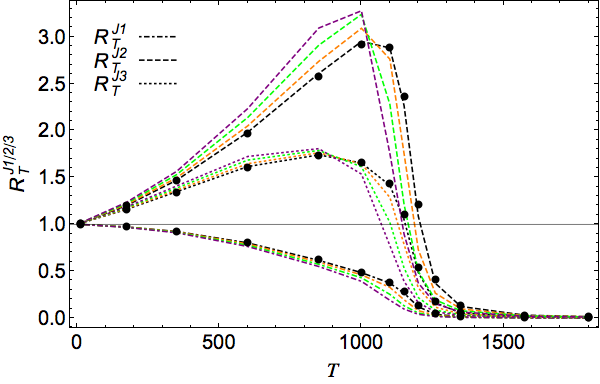

Supplement: Figure S1 — Flux responses in a network of three competitors: effect of large perturbations. Comparison of the flux response coefficients (towards changes in target concentration) calculated with expression (2) (depicted by the big dots), to the values obtained with the basic model (cf. Figure 3) for three competitors, simulated at different target levels (10-1800 molecules/cell). These response coefficients were derived from model simulations at different relative perturbations of the total target level: 1% (black lines), 5% (red lines), 10% (green lines), and 15% (purple lines), respectively. The two approaches give nearly identical results for the 1% perturbation and the difference increases gradually with larger perturbations. By analogy with the sigma factor example in the main text the total competitor levels were taken to be 700 molecules/cell for competitor 1 (σ70), 370 molecules/cell for competitor 2 (σ54) and 110 molecules/cell for competitor 3 (σ28). Other parameters needed for simulation are as in Figure 4A. (TIF) [file pone.0028494.s006.tif]

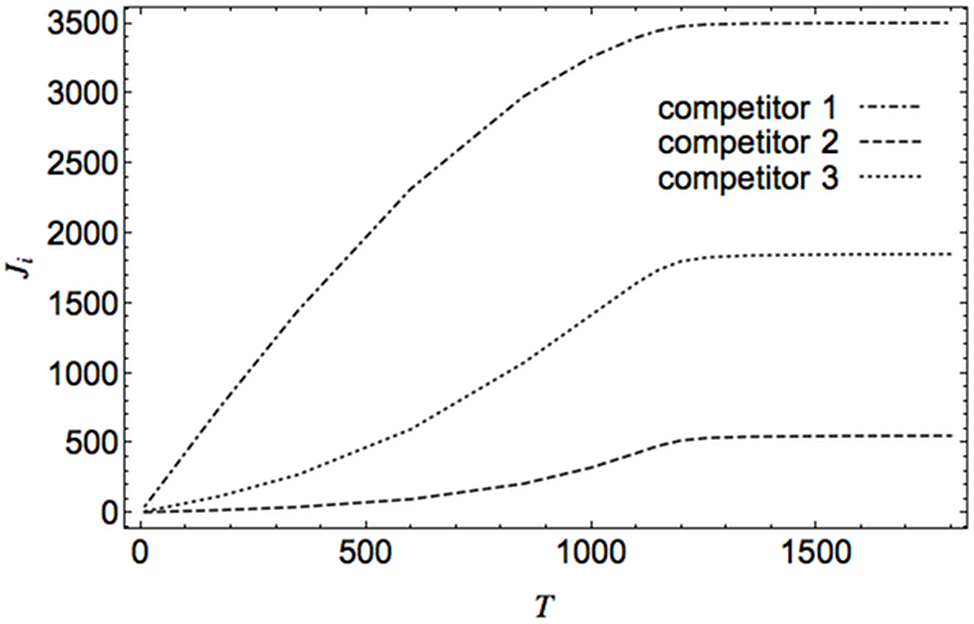

Supplement: Figure S2 — Input-output relations in a network of three competitors. Comparison of the fluxes (in molecules.min−1) obtained through simulation of the basic model (cf. Figure 3) for three competitors, simulated at different target levels (10-1800 molecules/cell). By analogy with the sigma factor example in the main text the total competitor levels were taken to be 700 molecules/cell for competitor 1 (σ70), 370 molecules/cell for competitor 2 (σ54) and 110 molecules/cell for competitor 3 (σ28). Other parameters needed for simulation were the reaction rate constants: kα1,f = 24, kα2,f = 8, kα3,f = 11 (molecules−1.min−1), kα1,r = 3, kα2,r = 6, kα3,r = 30 (min−1), and kβ1’ = kβ2’ = kβ3’ = 5 (min−1). The values were selected to fit the sigma factor example at a total target concentration of 700 molecules/cell (cf. Table 1). (TIF) [file pone.0028494.s007.tif]

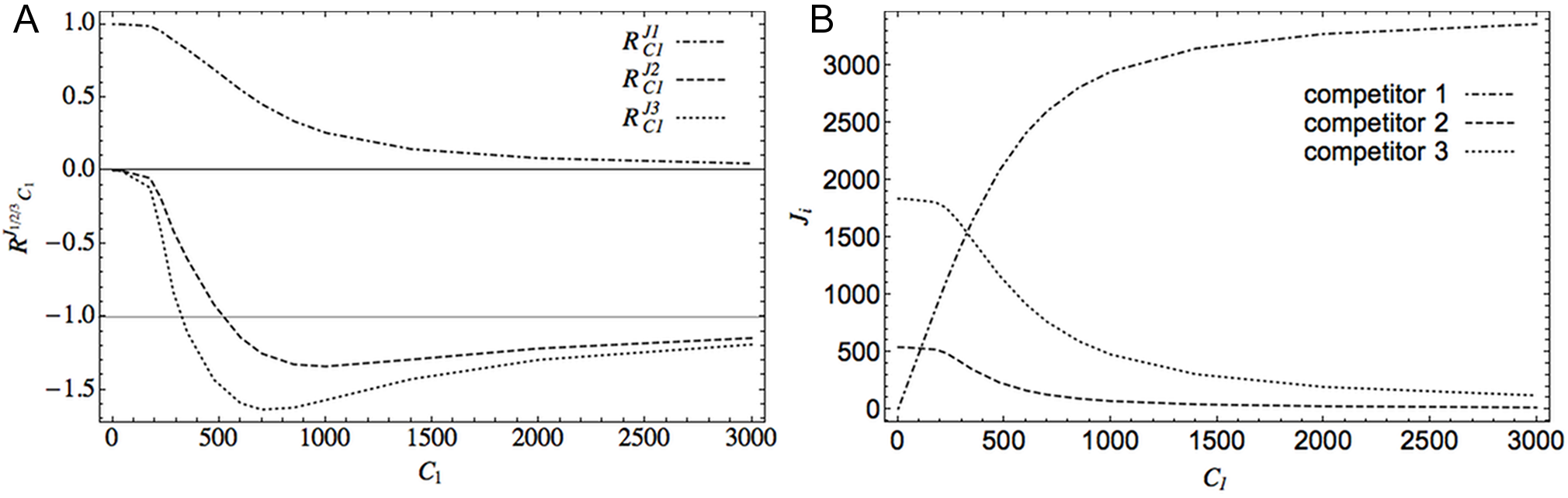

Supplement: Figure S3 — Input-output relations in a network of three competitors. (A). Flux response coefficients calculated for three competitors, simulated at different levels (1-3000 molecules/cell) of competitor 1. By analogy with the sigma factor example in the main text the total competitor levels that were fixed, were taken to be 370 molecules/cell for competitor 2 (σ54) and 110 molecules/cell for competitor 3 (σ28). The total target level was set at 700 molecules/cell. Other parameters needed for simulation were as in Figure S2. (B). Plot of input (total competitor level)/output (competitor flux in molecules.min−1) relation corresponding to (A). (TIF) [file pone.0028494.s008.tif]

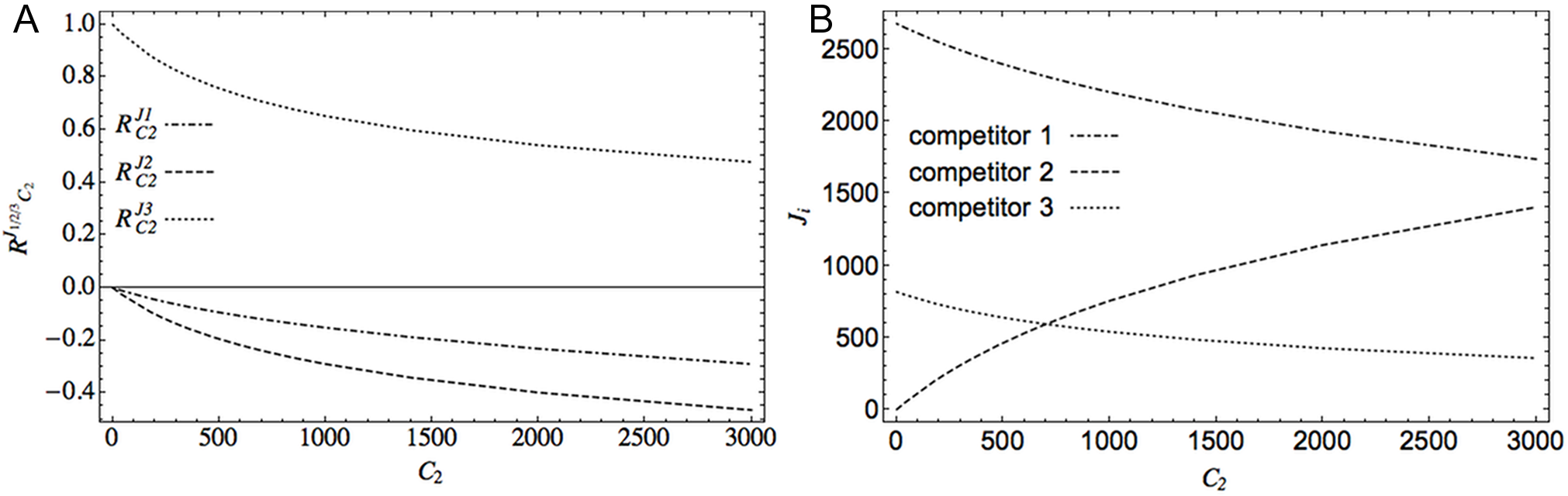

Supplement: Figure S4 — Input-output relations in a network of three competitors. (A). Flux response coefficients calculated for three competitors, simulated at different levels (1-3000 molecules/cell) of competitor 2. By analogy with the sigma factor example in the main text the total competitor levels that were fixed, were taken to be 700 molecules/cell for competitor 1 (σ70) and 110 molecules/cell for competitor 3 (σ28). The total target level was set at 700 molecules/cell. Other parameters needed for simulation were as in Figure S2. (B). Plot of input (total competitor level)/output (competitor flux in molecules.min−1) relation corresponding to (A). (TIF) [file pone.0028494.s009.tif]

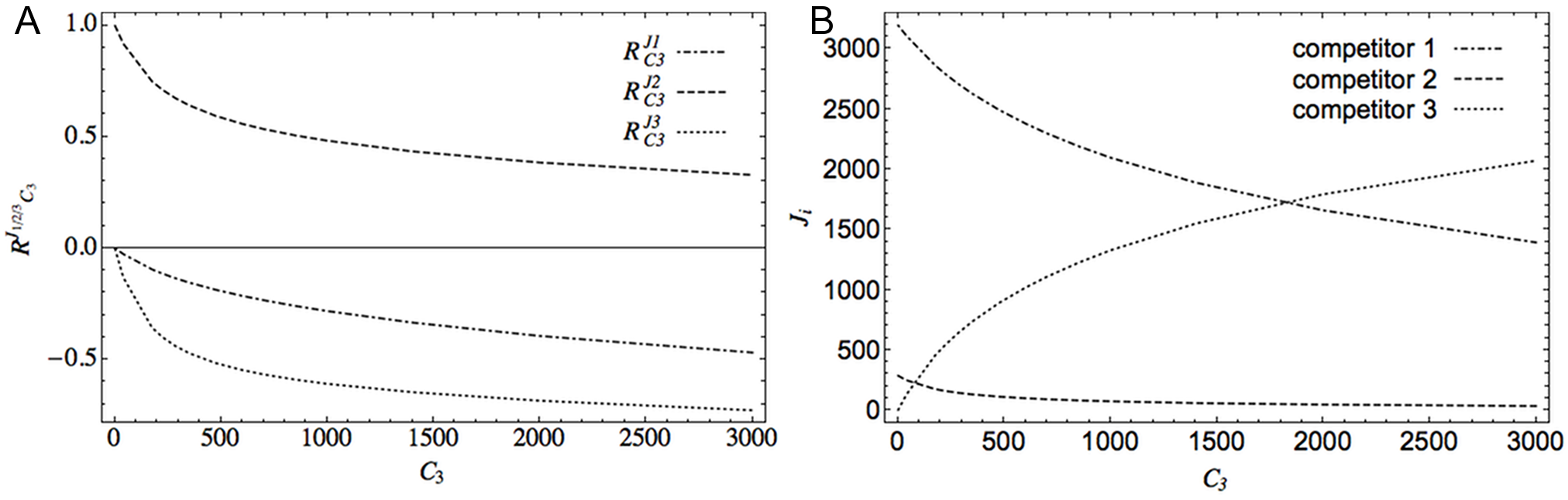

Supplement: Figure S5 — Input-output relations in a network of three competitors. (A). Flux response coefficients calculated for three competitors, simulated at different levels (1-3000 molecules/cell) of competitor 3. By analogy with the sigma factor example in the main text the total competitor levels that were fixed, were taken to be 700 molecules/cell for competitor 1 (σ70) and 370 molecules/cell for competitor 2 (σ54). The total target level was set at 700 molecules/cell. Other parameters needed for simulation were as in Figure S2. (B). Plot of input (total competitor level)/output (competitor flux in molecules.min−1) relation corresponding to (A). (TIF) [file pone.0028494.s010.tif]

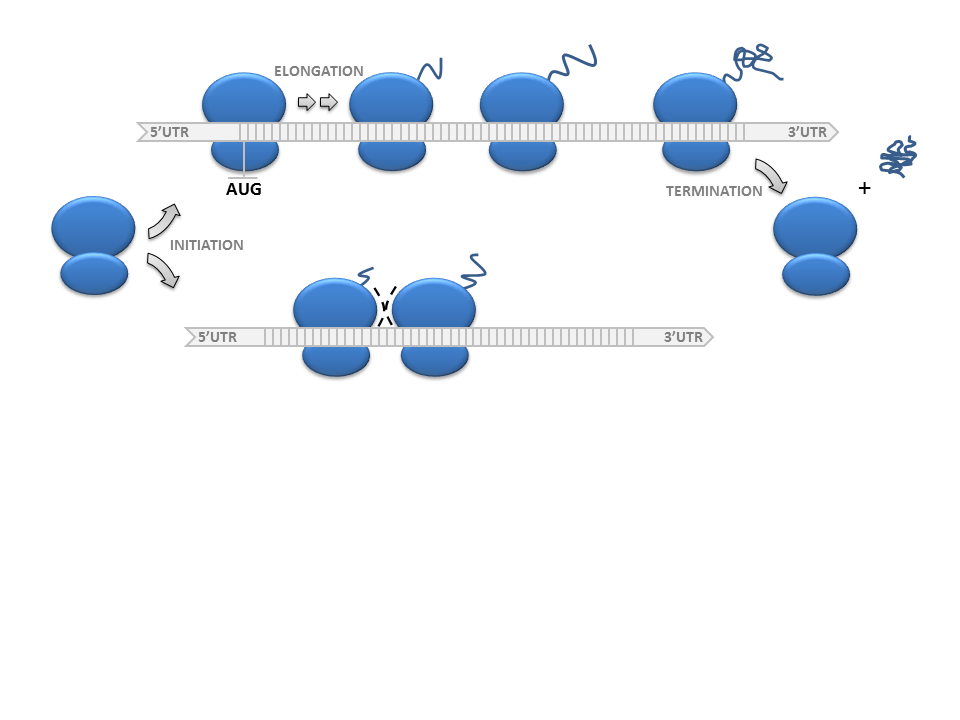

Supplement: Figure S6 — Translation model. The validity of expressions (8-10) was tested by comparison with a protein synthesis model based on Heinrich and Rapoport’s model [23]. This model takes into account the steric interactions of ribosomes as hard bodies occupying a fixed number of codons, moving along an mRNA template. Each state variable describes the (fractional) occupation by the front of a ribosome of an individual codon (the number of state variables is equal to the number of codons). Translation initiation is represented as an irreversible bimolecular reaction proportional to the available (free) ribosomes (subunit dissociation is not taken into account) and the fraction of free start sites (i.e. at the AUG codon with no sterically interacting ribosomes nearby). The translation elongation at a specific codon is represented by an irreversible unimolecular reaction proportional to the (fractional) occupation at that codon by the front of a ribosome. The corresponding rate constant is weighted by probability that the next codon is free to be occupied given that specific codon is occupied. The translation termination reaction is represented as an irreversible unimolecular reaction proportional to the (fractional) occupation of the last codon. To allow us to study competition the original model was extended to two mRNA templates as opposed to one. This means that three conservation relations exist i.e. for the total ribosome level and the total levels of the individual mRNAs. (TIF) [file pone.0028494.s011.tif]

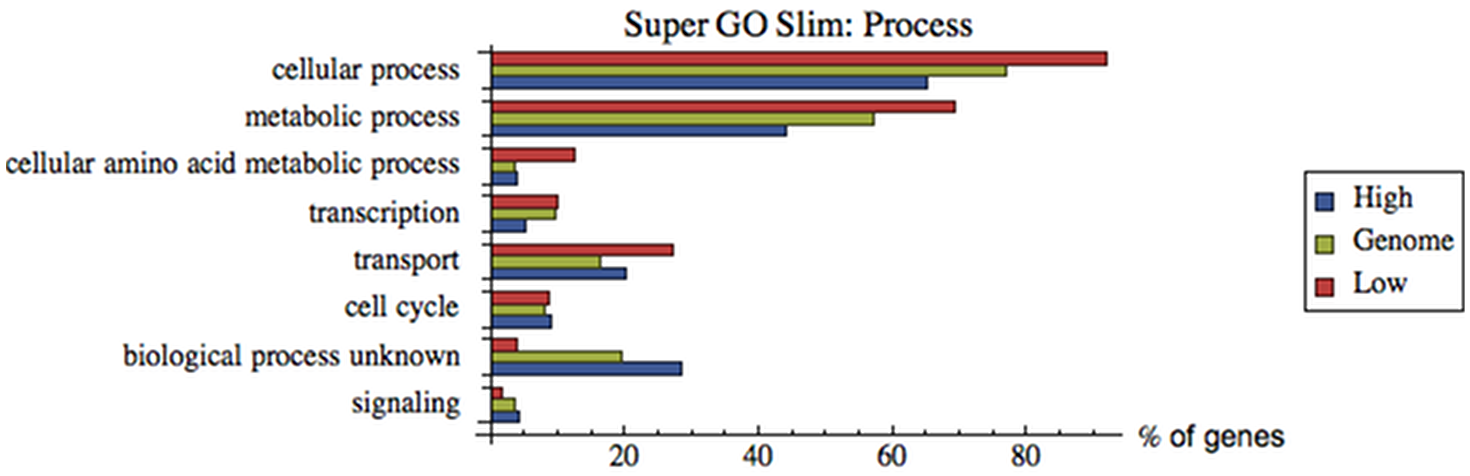

Supplement: Figure S7 — Gene Ontology mapping (in terms of high level biological process) of high and low response groups. Histogram indicating the relative frequency (as %) of GO classes in high and low response gene sets. The genes corresponding to mRNAs with the 5% highest (‘High’, blue bars) or lowest (‘Low’, red bars) response coefficients were pooled and mapped with the GO Slim Mapper tool (http://www.yeastgenome.org/cgi-bin/GO/goSlimMapper.pl), based on the ‘Super GO-Slim Process’ GO set. This is a small set of very broad, high level GO Biological Process terms, useful for binning groups of genes in general categories. The corresponding percentages for the whole yeast genome (as % of 6310 genes annotated at the moment of analysis, i.e. 4-3-2011, in the SGD) are represented by green bars (‘Genome’). (TIF) [file pone.0028494.s012.tif]

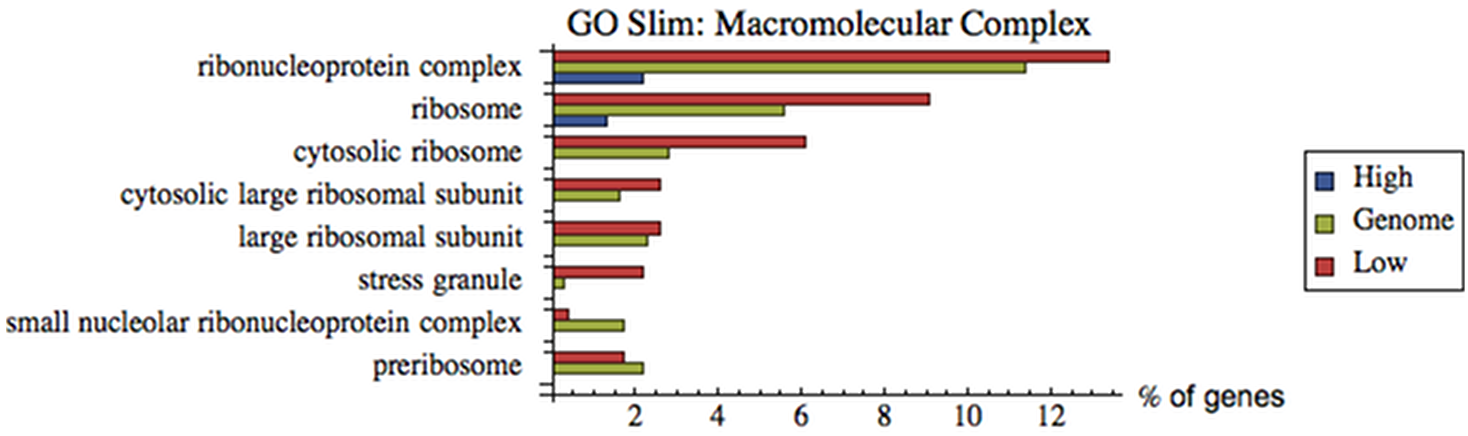

Supplement: Figure S8 — Gene Ontology mapping (in terms of molecular complex) of high and low response groups. Histogram indicating the relative frequency (as %) of GO classes in high and low response gene sets. The genes corresponding to the mRNAs with the 5% highest (‘High’, blue bars) and lowest (‘Low’, red bars) response coefficients were pooled and mapped with the GO Slim Mapper tool (http://www.yeastgenome.org/cgi-bin/GO/goSlimMapper.pl), based on the ‘Cellular Component’ ontology. This is a set of granular protein complex terms, useful for determining whether your protein of interest is a member of a particular complex. The corresponding percentages for the whole yeast genome (as % of 6310 genes annotated at the moment of analysis, i.e. 4-3-2011, in the SGD) are represented by green bars (‘Genome’). (TIF) [file pone.0028494.s013.tif]
